# Efficacy of a Smartphone-Based Digital Therapeutic (Anzeilax) in Generalized Anxiety Disorder: Randomized Controlled Trial

**DOI:** 10.2196/69981

**Published:** 2025-10-14

**Authors:** Chanmi Park, Hyunsil Song, Hanna Kim, Eunji Kim, Hye-Jeong Jo, Jae-Jin Kim, Jee Hang Lee, Jinwoo Kim

**Affiliations:** 1 Human Computer Interaction Lab Department of Cognitive Science Yonsei University Seoul Republic of Korea; 2 HAII Corp Seoul Republic of Korea; 3 Institute of Behavioral Science in Medicine Yonsei University College of Medicine Seoul Republic of Korea; 4 Department of Psychiatry Yonsei University College of Medicine Seoul Republic of Korea; 5 Department of Human-Centered AI Sangmyung University Seoul Republic of Korea; 6 Institute for Advanced Intelligence Study Daejeon Republic of Korea

**Keywords:** generalized anxiety disorder, randomized controlled trial, acceptance and commitment therapy, self-talk, digital therapeutic, mHealth, mobile health, digital health, mental health

## Abstract

**Background:**

Individuals with generalized anxiety disorder (GAD) often face challenges with self-regulation and limited access to traditional therapy. Although acceptance and commitment therapy (ACT) has demonstrated both efficacy and effectiveness in promoting psychological flexibility, scalable solutions are necessary to address these barriers. This study introduces Anzeilax, an ACT-based digital therapeutic (DTx) that incorporates self-talk as a novel mechanism of action (MoA) to enhance psychological flexibility in the treatment of GAD.

**Objective:**

This study aimed to evaluate the efficacy of Anzeilax in reducing anxiety symptoms in individuals with GAD.

**Methods:**

A 10-week, parallel-group, superiority randomized controlled trial (RCT) was conducted with 96 participants diagnosed with GAD (Generalized Anxiety Disorder 7-item scale [GAD-7] scores≥10, age≥19 years). The participants were randomly assigned (1:1) to receive either Anzeilax alongside treatment as usual (TAU, treatment group; n=48, 50%) or TAU alone (control group; n=48). Only the outcome evaluators were blinded to the group assignment. The primary outcome was the change in the GAD-7 score from baseline to week 10. The secondary outcomes included the Beck Anxiety Inventory (BAI) for anxiety symptoms, the Penn State Worry Questionnaire (PSWQ) for pathological worry, and the Hospital Anxiety and Depression Scale (HADS) for anxiety (HADS-A) and depression (HADS-D) symptoms. All self-report outcomes were assessed at baseline and at weeks 5 (midintervention), 10 (postintervention), and 15 (follow-up).

**Results:**

During the trial, 34 (71%) and 31 (65%) participants in the treatment group maintained at least 80% of the prescribed usage frequency at weeks 5 and 10, respectively. Based on the full analysis set (FAS), participants using Anzeilax demonstrated significant improvement in anxiety symptoms compared to the control group. Analysis of the primary outcome at 10 weeks postintervention compared to baseline exhibited a significant reduction in GAD-7 scores (adjusted mean difference –2.26, 95% CI –3.78 to –0.74, *P*=.002). Secondary outcomes at the same time point indicated consistent improvements, with significant group-by-time interactions observed in the GAD-7 (Cohen d=0.60, *P*=.008), BAI (Cohen d=0.50, *P*=.008), PSWQ (Cohen d=0.62, *P*=.002), and HADS-A (Cohen d=0.50, *P*=.01) scores. These improvements were sustained at the follow-up assessment (week 15). Although the differences in depressive symptoms between the two groups did not present statistical significance, notable improvements were observed in the treatment group.

**Conclusions:**

Anzeilax demonstrated clinically meaningful efficacy in reducing anxiety symptoms when combined with TAU. The results showed consistent improvements across multiple anxiety measures, with effects sustained through follow-up. The incorporation of context-sensitive self-talk within an ACT-based DTx framework offers a promising and accessible solution for treating individuals with GAD.

**Trial Registration:**

ClinicalTrials.gov NCT06010654; https://clinicaltrials.gov/study/NCT06010654

## Introduction

Generalized anxiety disorder (GAD) affects approximately 4% of the population [1,2] and is characterized by persistent and excessive worry that significantly disrupts daily functioning and diminishes quality of life [3,4]. The socioeconomic burden of GAD is substantial, comprising direct health care costs and reduced productivity [5]. Individuals with GAD often exhibit impaired self-regulation, which perpetuates excessive rumination and worry patterns [6]. This maladaptive cycle undermines their ability to adaptively engage with emotions across various contexts, whether positive, negative, or neutral [7,8].

Acceptance and commitment therapy (ACT) [9] has emerged as an effective intervention for GAD, which promotes psychological flexibility and cognitive defusion [10-12]. Recent meta-analyses have demonstrated ACT’s efficacy in anxiety disorders, with effect sizes comparable to traditional cognitive behavioral therapy (CBT) approaches [13,14]. This approach enables individuals to observe and disengage from distressing thoughts, while making value-aligned decisions [15]. However, traditional therapeutic approaches often encounter accessibility barriers due to logistical and scheduling constraints, emphasizing the need for scalable solutions [16]. The advent of smartphone-based digital therapeutics (DTxs) has addressed this gap by making ACT-based interventions more widely accessible [17,18]. The high smartphone ownership rates worldwide present an unprecedented opportunity to deliver therapeutic content directly to individuals in need [19]. In addition, several studies have demonstrated significant reductions in anxiety symptoms through these platforms [20-25].

DTxs for anxiety disorders have evolved considerably in recent years. For GAD, specifically, several platforms have demonstrated efficacy in randomized trials, with effect sizes ranging from small to moderate [26,27]. Although these digital interventions show promising potential, they naturally face some developmental challenges as the domain matures. For instance, many interventions are still in the process of establishing more robust theoretical foundations and working to enhance long-term engagement strategies. They are also gradually developing more sophisticated approaches to address the complex, context-dependent nature of anxiety symptoms [28-30]. Although some digital platforms effectively incorporate ACT principles into these sophisticated approaches [31], the discipline continues to explore innovative ways to translate the experiential and context-sensitive aspects that make in-person ACT delivery so effective into digital formats—a natural evolution as technology and therapeutic approaches continue to converge.

This study introduced *Anzeilax*, an innovative ACT-based DTx that incorporates self-talk as a mechanism of action (MoA) to enhance psychological flexibility and self-regulation. Self-talk—broadly defined as verbalizations or statements directed at oneself to serve self-regulatory functions [32]—is a central mechanism within self-regulation theory. It allows individuals to interpret situations, guide their behavior, and regulate emotions [33]. Past studies have demonstrated efficacy of self-talk in enhancing focus and self-regulation across various domains, including sports psychology and test anxiety [34,35].

As a mechanism for guiding thoughts and actions, self-talk plays a pivotal role in self-regulation; positive or constructive self-talk enhances focus and motivation, whereas negative or critical self-talk hinders performance [36]. Within the ACT framework, self-talk aligns with the principle of cognitive defusion, which encourages individuals to recognize thoughts as transient mental events, rather than absolute truths dictating behavior [37,38]. This approach transforms self-talk into supportive internal dialogue, helping individuals distance themselves from unhelpful thoughts and refocus on meaningful, value-driven action, effectively bridging self-regulation theory and ACT principles.

Anzeilax adopts self-talk in a context-sensitive manner, tailoring its application to the users’ emotional states. During negative emotional states, self-distancing strategies are used to reduce the intensity of rumination, promote psychological flexibility, and diminish the influence of maladaptive thought patterns [39]. Conversely, during positive states, self-referencing [38,39] techniques are incorporated to foster adaptive emotional engagement, thereby enhancing motivation and alignment with personal goals. By addressing the challenges individuals with GAD face in adaptive emotional processing [40], this context-sensitive approach bridges theoretical insights from ACT and self-regulation theory with practical, personalized interventions. This adaptivity addresses shortcomings of past DTxs, which typically use static intervention strategies regardless of the user’s emotional context [41].

We conducted a parallel-group, superiority randomized controlled trial (RCT) to compare the efficacy of Anzeilax combined with treatment as usual (TAU; treatment group) against TAU alone (control group) in individuals with GAD over a 10-week intervention period, with a follow-up assessment at week 15. Our primary hypothesis was that participants receiving Anzeilax combined with TAU would show a significantly greater reduction in GAD symptoms, as measured by the Generalized Anxiety Disorder 7-item scale (GAD-7) at the 10-week postintervention assessment compared to those receiving TAU alone. This hypothesis was based on the established efficacy of ACT for anxiety disorders [13] and evidence suggesting that digital delivery of psychological interventions can achieve clinically meaningful outcomes [42]. Secondary objectives included evaluating the effects of Anzeilax on specific anxiety symptoms, worry, and depression, both postintervention and at follow-up. Safety outcomes were also monitored to ensure the long-term sustainability of the intervention.

This study aimed to advance the field of DTxs for GAD by evaluating a novel, patient-centered approach that integrates ACT principles with context-sensitive self-talk. Through this innovative combination, Anzeilax seeks to offer an accessible and effective solution for managing GAD symptoms, potentially expanding access to evidence-based care [43].

## Methods

### Trial Design

A phase II, randomized (1:1), single-blind (evaluator-blind), parallel-group superiority trial was conducted with a primary endpoint at 10 weeks after randomization.

### Ethical Considerations

The Korea Ministry of Food and Drug Safety approved the trial protocol (protocol number 1289). The Institutional Review Board (IRB) of Gangnam Severance Hospital, Yonsei University, South Korea also approved the protocol (registration number 2022-0961-020; project number 3-2023-0018). The trial was registered at ClinicalTrials.gov (NCT06010654). The trial adhered to ethical standards in accordance with the Declaration of Helsinki. The study was conducted in full accordance with the pre-registered methodology, which ensured consistency and adherence to the original study design (see Multimedia Appendix 1 for the CONSORT (Consolidated Standards of Reporting Trials) E-HEALTH checklist of the trial).

All participants provided written informed consent before enrollment. The consent form clearly outlined their right to withdraw from the study at any time without penalty. The form ensured their full understanding of the study’s scope and procedures. It specified that their data could be used for both primary and secondary analyses. All participant data were deidentified prior to analysis in accordance with IRB-approved protocols to ensure privacy and confidentiality. Personally identifiable information (eg, names, contact details) was excluded from the analytic dataset. All participants received monetary compensation for their involvement. This included a participation fee of 20,000 KRW (US $15) for the first visit and a base fee of 10,000 KRW (US $7.50) each for the second, third, and fourth online assessments, which totaled up to South Korean won (KRW) 50,000, or US $37.50. Participants in the treatment group were also eligible for an additional compensation up to KRW 150,000 (US $112.50) based on their level of engagement with the intervention throughout the study.

### Participants

This clinical trial included adults aged 19 years or older with at least a high school diploma. Participants were required to have been diagnosed with GAD according to the *Diagnostic and Statistical Manual of Mental Disorders, Fifth Edition* (DSM-5), with *International Classification of Diseases 10th Revision* (ICD-10) code 300.02 (F41.1); have a GAD-7 score of 10 or higher (indicating moderate-to-severe anxiety); and to have been currently taking the prescribed medication for GAD. Individuals with other anxiety disorders, such as panic disorder (PD), social anxiety disorder (SAD), or major depressive disorder (MDD) accompanied by excessive worry (a key symptom of GAD), were also eligible. The participants had to fully understand the purpose, content, and process of the clinical trial and provide written consent to participate.

Participants were excluded if they met any of the following criteria: inability to read the consent form; lack of proficiency in using a smartphone; current or past psychiatric history (including schizophrenia, psychosis, bipolar disorder, or epilepsy); brain injury; cognitive impairment; neurological disorders; intellectual disability; substance or alcohol use disorder, suicidal intent, suicidal ideation, or self-harm within the past 6 months; recent (within the past 3 months) or current participation in CBT for anxiety, depression, or mood disorders; or enrollment in another clinical study. They were also excluded if the investigator deemed them unsuitable for the trial.

### Intervention

Participants in the treatment group were given access to the Anzeilax app, a self-guided DTx developed for iOS and Android platforms, and launched in 2023 by HAII Corp (see Multimedia Appendix 2). Anzeilax is designed for daily use over a 10-week intervention period and features two main programs: *Self-Talk* and *Self-Talk Plus*.

The primary program, Self-Talk, delivers one piece of content daily, accompanied by an introductory explanation to enhance user comprehension and engagement. Upon completing Self-Talk exercises, participants gain access to Self-Talk Plus, which allows them to express their current emotions by selecting descriptors, such as weather icons and emotion words. Based on these context inputs, the program generates customized self-talk exercises for participants to practice independently.

Both Self-Talk and Self-Talk Plus include two types of exercises: reading ACT-based metaphorical content and self-verbalization, which involves vocalizing responses to guided questions. These practices require users to record their responses while reading or answering prompts and listen to their recordings as a final reflective step, reinforcing the therapeutic content.

In addition to the main programs, users can access features such as log history, basic information, and reward badges through the app menu, providing additional motivation and tracking as they progress through the program.

To enhance user comfort during the listening phase, Anzeilax incorporates a voice equalization feature designed to address the common discomfort associated with hearing one’s recorded voice. Research on self-talk suggests that individuals often perceive their recorded voice as unfamiliar or uncomfortable due to the absence of the bone conduction component in the sound [44-46]. The voice equalization feature mitigates this discomfort by adjusting the audio frequencies: amplifying mid- and high-frequency bands, while attenuating low-, low-mid-, and ultrahigh-frequency bands. This process creates a more natural and pleasant auditory experience for users.

To promote consistent engagement, automated push notifications were tailored to adapt to individual usage patterns. Participants were instructed to use Anzeilax at least 4 days per week throughout the 10-week intervention period. If a participant’s usage indicated a risk of falling below this threshold, a reminder notification with an encouraging message was sent. For participants who maintained regular usage, motivational messages were delivered to reinforce their engagement.

The frequency and content of these notifications were carefully calibrated to avoid overwhelming participants with repetitive or excessive messaging, ensuring sustained adherence to the program without causing notification fatigue.

### Study Procedure and Randomization

To facilitate recruitment, the RCT was publicized through multiple channels, including Seoul Metro advertisements, hospital postings, and psychiatric patient community websites. Those interested applied for the trial using Google Forms. After an initial online screening, eligible participants underwent a video interview via Zoom with a certified clinical psychologist using the Mini-International Neuropsychiatric Interview [47] to confirm the diagnosis and inclusion criteria. To ensure the accuracy of diagnoses, only patients with a confirmed diagnosis of GAD were recruited for this study. As part of the screening process, participants were required to present their recent prescription records, which were reviewed by the certified clinical psychologist to verify their diagnosis. This approach helped maintain diagnostic validity and consistency across the study sample.

Prior to the trial, participants received verbal and written explanations of the study procedures and provided written informed consent during a face-to-face baseline assessment meeting at Gangnam Severance Hospital. Allocation numbers were generated by the sponsor, sealed in opaque envelopes, and provided to the researchers. Upon meeting eligibility requirements and providing consent, each participant was randomly assigned to either the treatment group (Anzeilax and TAU) or the control group (TAU only) by drawing an envelope containing their allocation number.

Each participant drew a slip marked “treatment group” or “control group” under the supervision of the clinical research coordinator, who documented the randomization date and signed the slip. The randomization number, along with the participant’s screening number, served as the identification code throughout the trial. As a single-blind (evaluator-blind) study, the randomization records were managed by the principal investigator and a designated randomization officer.

For participants assigned to the treatment group, personal login codes for Anzeilax were provided immediately after the baseline assessment, ensuring that intervention initiation occurred without delay. Participants were instructed to use the app at least four times per week over the 10-week intervention period. Although participants could flexibly access the app based on their schedules, engagement was encouraged through an incentive system. Assessments for both groups were conducted at weeks 5, 10, and 15, with all postbaseline assessments administered online.

TAU, applied to both groups, consisted of maintaining participants’ existing treatment regimens, including current psychiatric medications. Medication management followed the pharmacological treatment guidelines for GAD established by the Korean Medical Association. Adjustments to medication type or dosage, as deemed necessary by the treating psychiatrist, were allowed and not restricted by the study protocol.

TAU was provided at different hospitals, ensuring that the psychiatrists providing TAU were unaware of their patients’ participation in the study, thereby minimizing potential bias. This ensured that TAU was prescribed independently of the research, maintaining standard care without influence from the study protocol or trial considerations.

### Sample Size

A sample size of 66 participants (n=33, 50%, per group) was required to detect a small-to-medium effect size (Cohen d=0.5) with 80% power and a one-sided significance level (α=.025, Z=1.960) between the groups [48-50]. Accounting for an anticipated 30% attrition rate (19%-45%) [21,36,37], an additional 15 participants per group were included, resulting in a total sample size of 96 participants (n=48, 50%, per group).

### Measures

Assessments were conducted at four time points: baseline (week 0), midintervention (week 5), postintervention (week 10), and follow-up (week 15). Baseline measurements were completed in person at the hospital, while subsequent assessments were conducted online. The primary outcome measure was the GAD-7 [38], a validated tool for measuring the severity of GAD symptoms. The GAD-7 consists of 7 items rated on a 4-point Likert scale (0-3), with a total score range of 0-21. Based on the score, anxiety severity is classified as minimal (0-4), mild (5-9), moderate (10-14), or severe (15-21). In this study, a GAD-7 score of 10 or higher was used as the cutoff for determining clinically significant anxiety.

Secondary outcomes included specific domains of anxiety, evaluated using standardized measures. Cognitive and physical symptoms of anxiety were assessed with the Beck Anxiety Inventory (BAI) [51], worry severity was measured using the Penn State Worry Questionnaire (PSWQ) [52], and overall anxiety and depression symptoms were evaluated using the Hospital Anxiety and Depression Scale (HADS; HADS-A for anxiety and HADS-D for depression) [53]. More detailed information on the cutoff scores can be found in Section 5 in Multimedia Appendix 2.

Participant safety was monitored throughout the study period via the spontaneous reporting of adverse events, defined as any undesirable clinical or physical findings not present before the trial began, including anticipated side effects. Reported adverse events were classified as mild, moderate, or severe based on their severity.

To evaluate participants’ engagement and experience, adherence to the intervention was measured, offering valuable insights into their response to the Anzeilax DTx and TAU. This metric was crucial for assessing engagement levels and user satisfaction throughout the intervention period.

### Statistical Analysis

The analysis was conducted using the full analysis set (FAS) with adjustments made using the last observation carried forward (LOCF) method. Subsequently, analysis was performed on the per protocol (PP) population. In cases where discrepancies were observed between the FAS and PP results, the FAS analysis was designated as the primary analysis method, with PP analysis serving as a supplementary approach. To assess consistency, the results of the PP analysis were compared with those of the FAS analysis.

For the primary outcome analysis, GAD-7 scores were collected through surveys at the first visit (baseline) and after completion (week 10). For the treatment group, the values before (baseline) and after using Anzeilax (week 10) were summarized using descriptive statistics. For the control group, the values before starting TAU (baseline) and after 10 weeks were summarized similarly. For the primary efficacy endpoint analysis of the GAD-7 scores, a between-group comparison of the difference in the scores from baseline to week 10 was performed using ANCOVA, with baseline values as a covariate. The significance level was set at a one-sided *P* value of <.03.

To evaluate the secondary outcomes, changes over time, and between-group differences in symptom relief compared to baseline for the treatment and control groups, repeated-measures ANOVA was conducted at 5-week intervals over a total of 15 weeks (10-week intervention and 5-week follow-up periods). The analysis was performed on the GAD-7, BAI, PSWQ, and HADS measurements taken at baseline, midintervention (5 weeks), postintervention (10 weeks), and follow-up (15 weeks). The significance level was set at a one-sided *P* value of <.03.

All statistical analyses were conducted using R (version 4.4.1; R Foundation for Statistical Computing), an open source statistical computing software program, and each participant was assigned a unique study-specific identifier. Additional statistical analyses, including alternative missing data–handling methods and sensitivity analyses, were conducted to assess the robustness of our findings. The details of these analyses and their results are provided in Sections 7-11 in Multimedia Appendix 2.

## Results

### Baseline Characteristics

Between August 14, 2023, and April 11, 2024, 2034 people were screened for the trial. Of these, 1723 (84.7%) were excluded because their anxiety levels were lower than 10 on the GAD-7. A total of 311 (15.3%) patients were assessed for eligibility, and 96 (30.9%) met all the inclusion criteria and were randomized to either the treatment (n=48, 50%) or the control (n=48, 50%) group; of these, 61 (63.5%) were females. The mean age was 30 (SD 7.87) years, with a range of 19-60 years. Table 1 provides an overview of the demographics and baseline scores for primary and secondary outcomes by group. The postintervention and follow-up assessments were completed on June 20 and July 25, 2024, respectively.

**Figure 1:**
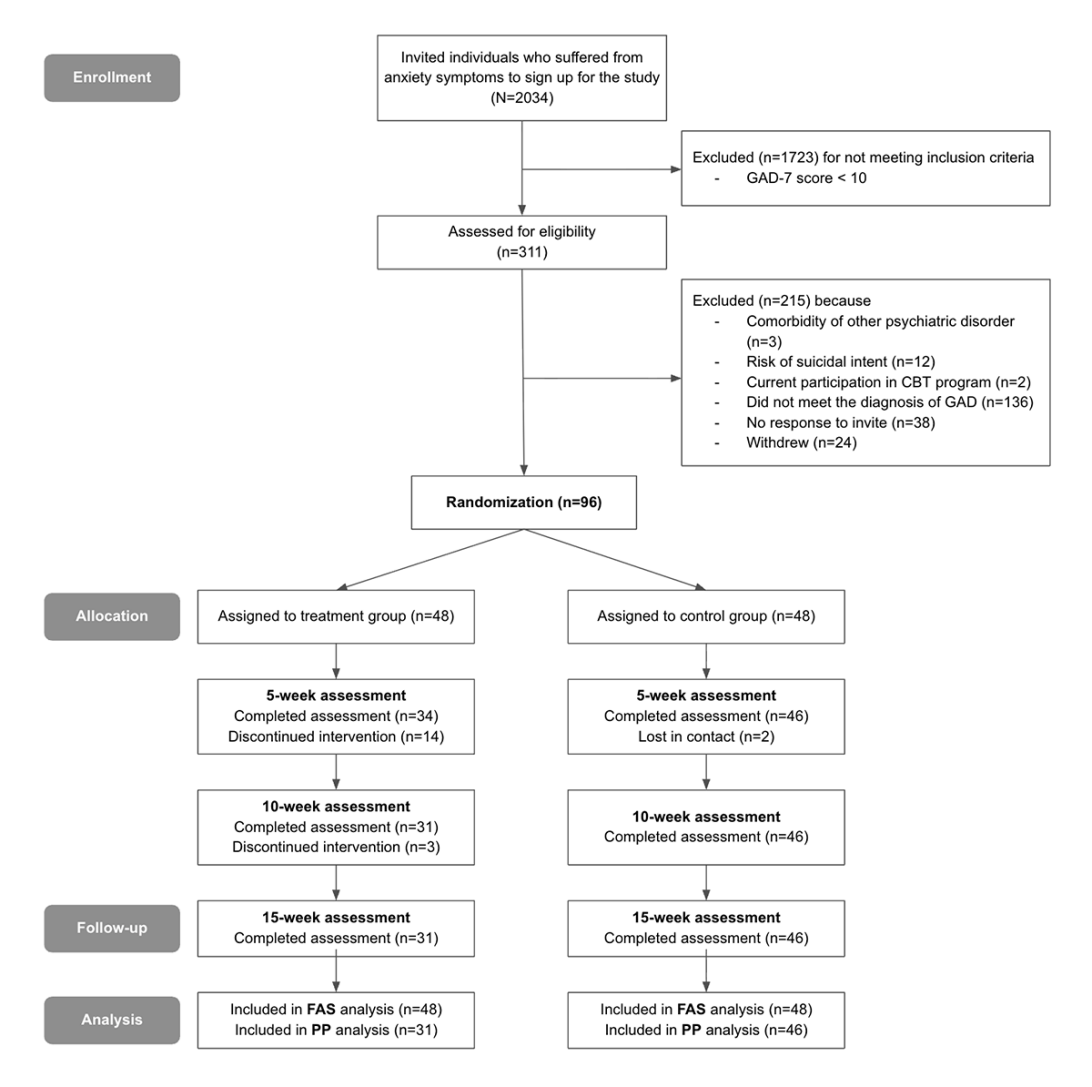
CONSORT flow diagram of participants. CBT: cognitive behavioral therapy; CONSORT: Consolidated Standards of Reporting Trials; FAS: full analysis set; GAD: generalized anxiety disorder; GAD-7: Generalized Anxiety Disorder 7-item scale; PP: per protocol.

In the treatment group, 17 (35.4%) participants discontinued the intervention. Among those who completed the trial, the participants practiced an average of 63 and 81 Self-Talk and Self-Talk Plus sessions, respectively. The frequency of Self-Talk Plus practice ranged from 46 to 243. Self-verbalization, which involves vocalizing responses to guided questions, was the most frequently selected program type in Self-Talk Plus (reading ACT content: 1029; self-verbalization: 1467). In the control group, 2 (4.2%) participants were lost to follow-up. Thus, 77 (80.2%) participants (treatment arm: n=31, 40.3%; control arm: n=46, 59.7%) completed all the trial assessments (Figure 1).

**Table 1:** Baseline characteristics of study participants (N=96).

		Overall (N=96)	Treatment arm (n=48)	Control arm (n=48)	*χ*^2^ (*df*)		*P* value	
Age (years), mean (SD)		30.00 (7.87)	31.92 (8.78)	28.92 (6.59)	—^a^		—	
**Gender, n (%)**						0.45 (1)		.83
	Male	35 (36.5)	17 (35.4)	18 (37.5)	—		—	
	Female	61 (63.5)	31 (64.6)	30 (62.5)	—		—	
**Education, n (%)**						3.21 (3)		.36
	Doctoral degree	1 (1)	0	1 (2.1)	—		—	
	Master’s degree	10 (10.4)	6 (12.5)	4 (8.3)	—		—	
	Undergraduate degree	59 (61.5)	32 (66.7)	27 (56.3)	—		—	
	High school	26 (27.1)	10 (20.8)	16 (33.3)	—		—	
**Work status, n (%)**						5.28 (2)		.71
	Employed	49 (51.0)	30 (62.5)	19 (39.6)	—		—	
	Not employed	43 (44.8)	16 (33.3)	27 (56.2)	—		—	
	Did not answer	4 (4.2)	2 (4.2)	2 (4.2)	—		—	
**Anxiety history (years), n (%)**						2.39 (3)		.50
	<1	8 (8.3)	3 (6.3)	5 (10.4)	—		—	
	1-5	57 (59.4)	30 (62.5)	27 (56.3)	—		—	
	6-10	22 (22.9)	9 (18.8)	13 (27.1)	—		—	
	>10	9 (9.4)	6 (12.5)	3 (6.3)	—		—	
**Psychiatric comorbidities, n (%)**						—		—
	MDD^b^	50 (52.1)	27 (56.3)	23 (47.9)	0.67 (1)		.41	
	PD^c^	25 (26)	8 (16.7)	17 (35.4)	4.38 (1)		.04	
	SAD^d^	3 (3.1)	1 (2.1)	2 (4.2)	0.34 (1)		.56	
	Other	23 (24.0)	8 (16.7)	15 (31.3)	2.8 (1)		.09	
Physical comorbidities, n (%)		21 (21.9)	9 (18.8)	12 (25.0)	0.55 (1)		.46	
**Outcomes, mean (SD)**								
	Baseline GAD-7^e^	12.36 (3.49)	12.42 (3.57)	12.31 (3.45)	16.7 (14)		.27	
	Baseline BAI^f^	25.10 (10.02)	24.81 (11.4)	25.40 (8.54)	50.1 (36)		.06	
	Baseline PSWQ^g^	56.44 (4.7)	57.23 (4.64)	55.65 (4.68)	22.6 (20)		.31	
	Baseline HADS-A^h^	11.59 (3.81)	13.44 (3.67)	13.19 (2.63)	18.2 (15)		.25	
	Baseline HADS-D^i^	13.31 (3.18)	12.19 (4.10)	11.00 (3.43)	21.4 (16)		.16	

^a^Not applicable.

^b^MDD: major depressive disorder.

^c^PD: panic disorder.

^d^SAD: social anxiety disorder.

^e^GAD-7: Generalized Anxiety Disorder 7-item scale.

^f^BAI: Beck Anxiety Inventory.

^g^PSWQ: Penn State Worry Questionnaire.

^h^HADS-A: Hospital Anxiety and Depression Scale – Anxiety.

^i^HADS-D: Hospital Anxiety and Depression Scale – Depression.

### Intervention Effects on the Primary Outcome

To ensure the robustness of the findings, we examined the potential impact of outliers. No data points were excluded solely based on outlier detection, as the study followed the intention-to-treat (ITT) principle within the FAS. Consequently, all randomized participants were included in the analysis, and no extreme values were identified that warranted exclusion.

The FAS and PP groups demonstrated reductions in GAD-7 scores from baseline to postintervention (week 10). In the FAS population, the treatment group’s mean GAD-7 score decreased from 12.42 (SD 3.57) at baseline to 8.83 (SD 3.99) at week 10, reflecting a mean reduction of 3.58 points (95% CI –4.88 to –2.28). In comparison, the control group’s mean GAD-7 score decreased from 12.31 (SD 3.45) at baseline to 11.04 (SD 4.29) at week 10, indicating a mean reduction of 1.27 points (95% CI –2.35 to –0.19). In the PP population, the treatment group (n=31, 64.6%) exhibited a mean reduction of 4.94 points (95% CI –6.52 to –3.36), with mean scores decreasing from 12.10 (SD 3.49) at baseline to 7.16 (SD 3.40) at week 10. However, the control group (n=46, 95.8%) showed a mean reduction of 1.33 points (95% CI –2.45 to –0.21), with mean scores declining from 12.37 (SD 3.49) at baseline to 11.04 (SD 4.37) at week 10 (Table 2).

**Figure 2:**
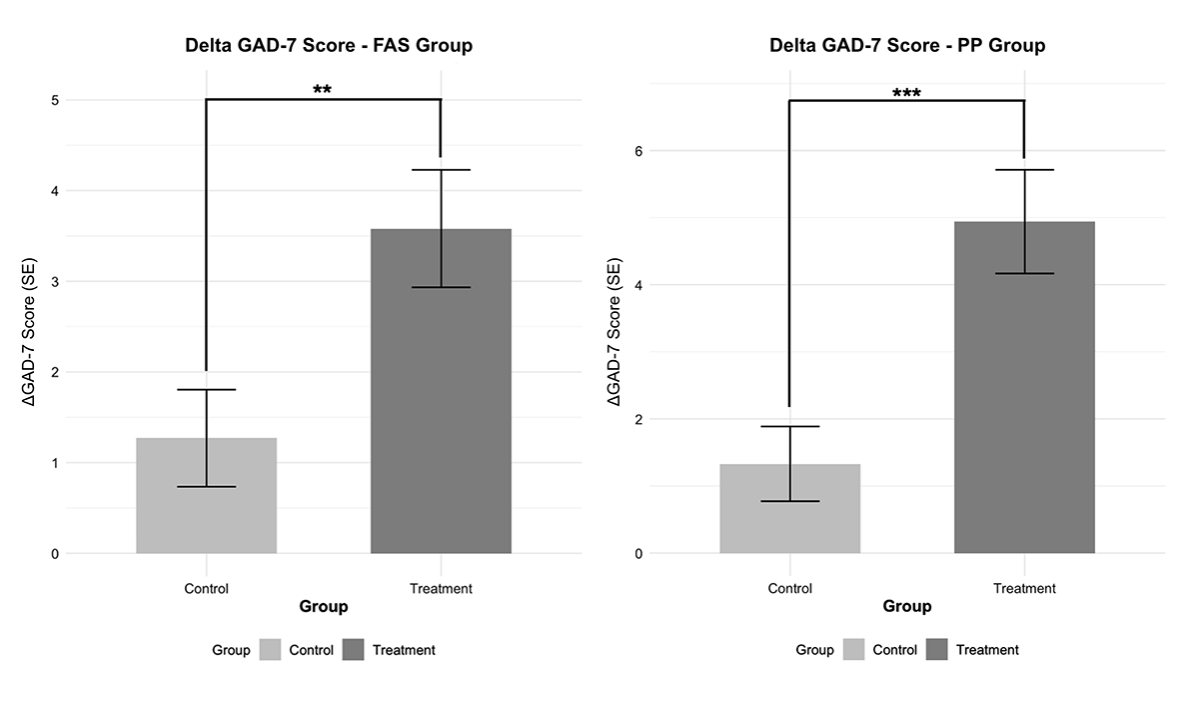
Comparison of the adjusted mean difference in GAD-7 scores between the treatment and control groups at week 10 in the FAS and PP populations. FAS: full analysis set; PP: per protocol; GAD: generalized anxiety disorder; GAD-7: Generalized Anxiety Disorder 7-item scale.

The analysis of treatment effects using ANCOVA, controlling for baseline scores, revealed significant differences between the groups in both analysis sets (Figure 2). In the FAS population, the adjusted mean difference between groups was –2.26 points (95% CI –3.78 to –0.74, *P*=.002), with a medium effect size (Cohen d=0.60). The PP analysis showed an even more pronounced treatment effect, with an adjusted mean difference of –3.74 points (95% CI –5.41 to –2.07, *P*<.001) and a large effect size (Cohen d=0.95). See Table 3 and Multimedia Appendix 2.

**Table 2:** Changes in GAD-7^a^ scores from baseline to postintervention (week 10) in FAS^b^ and PP^c^ populations.

Population			Baseline, mean (SD)		Postintervention, mean (SD)		Change in mean (95% CI)
**FAS**							
	Treatment group (n=48)	12.42 (3.57)		8.83 (3.99)		–3.58 (–4.88 to –2.28)	
	Control group (n=48)	12.31 (3.45)		11.04 (4.29)		–1.27 (–2.35 to –0.19)	
**PP**							
	Treatment group (n=31)	12.10 (3.49)		7.16 (3.40)		–4.94 (–6.52 to –3.36)	
	Control group (n=46)	12.37 (3.49)		11.04 (4.37)		–1.33 (–2.45 to –0.21)	

^a^GAD-7: Generalized Anxiety Disorder 7-item scale.

^b^FAS: full analysis set.

^c^PP: per protocol.

**Table 3:** Postintervention outcomes for GAD-7^a^ in FAS^b^ and PP^c^ populations.

Population	Adjusted difference, mean (SE; 95% CI)	*P* value	Effect size (Cohen d)
FAS (treatment vs control)	–2.26 (0.77; –3.78 to –0.74)	.002	0.60
PP (treatment vs control)	–3.74 (0.84; –5.41 to –2.07)	<.001	0.95

^a^GAD-7: Generalized Anxiety Disorder 7-item scale.

^b^FAS: full analysis set.

^c^PP: per protocol.

### Intervention Effects on Secondary Outcomes

**Figure 3:**
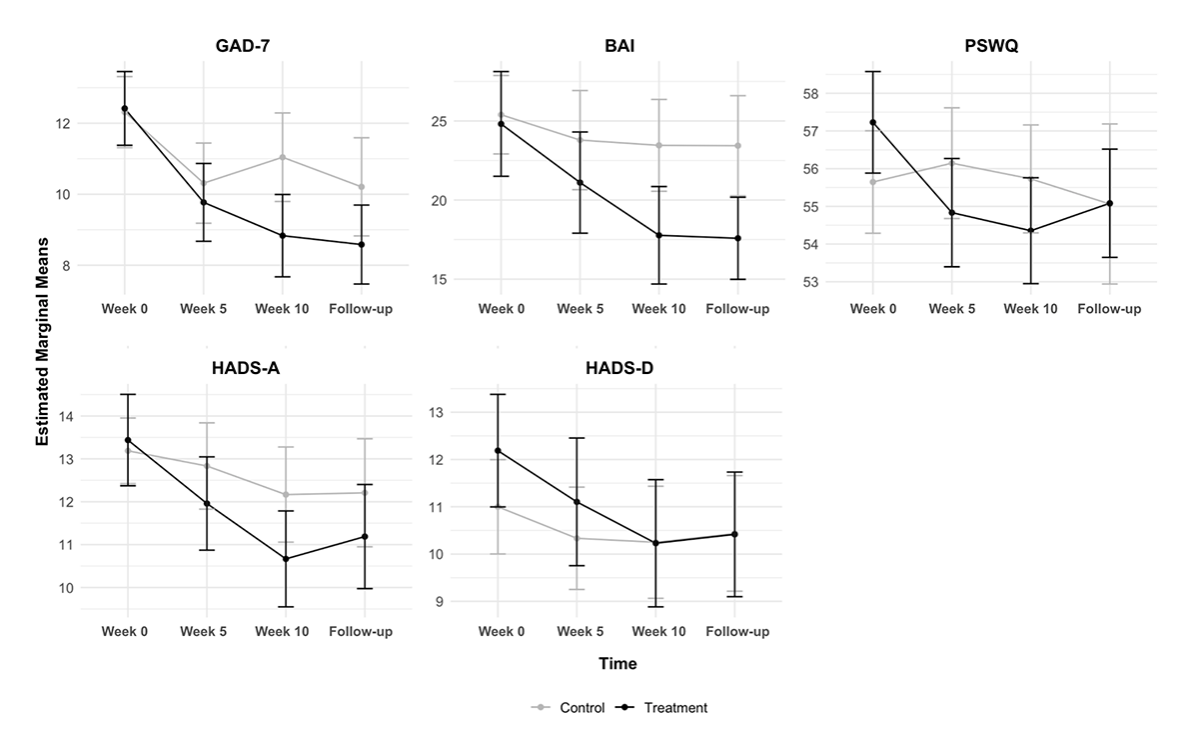
Mean changes in GAD-7, BAI, PSWQ, HADS-A, and HADS-D scores between treatment and control groups in the FAS population. BAI: Beck Anxiety Inventory; FAS: full analysis set; GAD: generalized anxiety disorder; GAD-7: Generalized Anxiety Disorder 7-item scale; HADS-A: Hospital Anxiety and Depression Scale – Anxiety; HADS-D: Hospital Anxiety and Depression Scale – Depression; PSWQ: Penn State Worry Questionnaire.

The secondary outcome measures, including GAD-7, BAI, PSWQ, and HADS subscales for anxiety and depression, were analyzed using repeated-measures ANOVA to examine group-by-time interactions across four time points: baseline, midintervention (week 5), postintervention (week 10), and follow-up (week 15). See Table 4 and Figure 3.

**Table 4:** Impact of the intervention on secondary outcome measures (FAS^a^).

Outcome measure		Treatment group, mean (SD)	Control group, mean (SD)	Estimated mean change difference, mean (95% CI)	*P* value (group×time)	Effect size (Cohen d)
**GAD-7^b^**						
	Baseline	12.42 (3.57)	12.31 (3.45)	0.1 (–1.34 to 1.55)	—^c^	—
	Midintervention (week 5)	9.77 (3.78)	10.31 (3.89)	–0.54 (–2.12 to 1.03)	.25	—
	Postintervention (week 10)	8,83 (3.99)	11.04 (4.29)	–2.21 (–3.91 to –0.51)	.008	0.60
	Follow-up (week 15)	8.58 (3.83)	10.21 (4.76)	–1.62 (–3.4 to 0.15)	.009	—
**BAI^d^**						
	Baseline	24.81 (11.4)	25.40 (8.54)	–0.58 (–4.72 to 3.55)	—	—
	Midintervention (week 5)	21.10 (11.0)	23.79 (10.8)	–2.69 (–7.17 to 1.79)	.12	—
	Postintervention (week 10)	17.77 (10.6)	23.46 (9.99)	–5.69 (–9.92 to –1.45)	.008	0.50
	Follow-up (week 15)	17.58 (8.93)	23.44 (10.9)	–5.85 (–9.95 to –1.76)	.004	—
**PSWQ^e^**						
	Baseline	57.23 (4.64)	55.65 (4.68)	1.58 (–0.33 to 3.5)	—	—
	Midintervention (week 5)	54.83 (4.94)	56.15 (5.06)	–1.31 (–3.37 to 0.74)	.10	—
	Postintervention (week 10)	54.35 (4.84)	55.73 (4.93)	–1.38 (–3.38 to 0.63)	.002	0.62
	Follow-up (week 15)	55.08 (4.94)	55.06 (7.31)	0.02 (–2.54 to 2.58)	.007	—
**HADS-A^f^**						
	Baseline	13.44 (3.67)	13.19 (2.63)	0.25 (–1.06 to 1.56)	—	—
	Midintervention (week 5)	11.96 (3.75)	12.83 (3.47)	–0.88 (–2.36 to 0.61)	.10	—
	Postintervention (week 10)	10.67 (3.85)	12.17 (3.82)	–1.5 (–3.08 to 0.08)	.01	0.50
	Follow-up (week 15)	11.19 (4.18)	12.21 (4.34)	–1.02 (–2.77 to 0.73)	.04	—
**HADS-D^g^**						
	Baseline	12.19 (4.10)	11.00 (3.43)	1.19 (–0.36 to 2.74)	—	—
	Midintervention (week 5)	11.10 (4.65)	10.33 (3.73)	0.77 (–0.96 to 2.5)	.03	—
	Postintervention (week 10)	10.23 (4.63)	10.25 (4.08)	–0.02 (–1.81 to 1.77)	.10	0.30
	Follow-up (week 15)	10.42 (4.54)	10.44 (4.21)	–0.02 (–1.82 to 1.78)	.10	—

^a^FAS: full analysis set.

^b^GAD-7: Generalized Anxiety Disorder 7-item scale.

^c^Not applicable.

^d^BAI: Beck Anxiety Inventory.

^e^PSWQ: Penn State Worry Questionnaire.

^f^HADS-A: Hospital Anxiety Depression Scale – Anxiety.

^g^HADS-D: Hospital Anxiety Depression Scale – Depression.

The analysis of GAD-7 scores revealed a significant group-by-time interaction through postintervention (*F*_1.95, 183.60_=4.32, *P*=.008) and follow-up (*F*_2.78, 261.77_=3.51, *P*=.009). The treatment group demonstrated greater reductions from baseline (mean 12.42, SD 3.57) to week 10 (mean 8.83, SD 3.99) compared to controls from baseline (mean 12.31, SD 3.45) to week 10 (mean 11.04, SD 4.29), with a medium effect size (Cohen d=0.60). The estimated mean change differences between groups for GAD-7 scores were –0.54 (95% CI –2.12 to 1.03) at week 5, –2.21 (95% CI –3.91 to –0.51) at week 10, and –1.62 (95% CI –3.4 to 0.15) at week 15. Both groups showed initial reductions through midintervention (week 5); however, the trajectory diverged thereafter. Although the treatment group continued to show decreasing GAD-7 scores, the control group demonstrated a rebound effect after week 5, followed by a slight reduction postintervention.

The analysis of anxiety symptoms using the BAI revealed a significant group-by-time interaction through postintervention (*F*_1.99, 187.11_=4.22, *P*=.008) and follow-up (*F*_2.83, 266.25_=4.17, *P*=.004). Despite similar baseline scores in the treatment group (mean 24.81, SD 11.4) and control group (mean 25.40, SD 8.54), the treatment group showed substantial reductions through week 10 (mean 17.77, SD 10.6), which were maintained at week 15 (mean 17.58, SD 8.93). The control group showed minimal changes from baseline (mean 25.40, SD 8.54) to week 10 (mean 23.46, SD 9.99) and week 15 (mean 23.44, SD 10.9). The estimated mean change difference between groups increased from week 10 (–5.69, 95% CI –9.92 to 1.45) to week 15 (–5.85, 95% CI –9.95 to –1.76), indicating a growing treatment effect over time. The change-based effect size was medium (Cohen d=0.50).

The PSWQ showed a significant group-by-time interaction through postintervention (*F*_1.92, 180.93_=6.26, *P*=.002) and follow-up (*F*_2.76, 259.84_=3.71, *P*=.007). The treatment group demonstrated reductions from baseline (mean 57.23, SD 4.64) to week 10 (mean 54.35, SD 4.84), whereas control group scores remained stable from baseline (mean 55.65, SD 4.68) to week 10 (mean 55.73, SD 4.93), yielding a medium effect size (Cohen d=0.62). The estimated mean change differences between groups were –1.31 (95% CI –3.37 to 0.74) at week 5, –1.38 (95% CI –3.38 to 0.63) at week 10, and 0.02 (95% CI –2.54 to 2.58) at week 15.

HADS-A showed significant group-by-time interactions through postintervention (*F*_1.96, 183.96_=3.66, *P*=.014) and follow-up (*F*_2.69, 253.13_=2.36, *P*=.039). The treatment group demonstrated greater reductions from baseline (mean 13.44, SD 3.67) to week 10 (mean 10.67, SD 3.85) compared to controls from baseline (mean 13.19, SD 2.63) to week 10 (mean 12.17, SD 3.82), with a medium effect size (Cohen d=0.50). The estimated mean change differences between groups for HADS-A were –0.88 (95% CI –2.36 to 0.61) at week 5, –1.5 (95% CI –3.08 to 0.08) at week 10, and –1.02 (95% CI –2.77 to 0.73) at week 15. The significant group difference observed postintervention indicated a greater reduction in HADS-A scores for the treatment group compared to the control group.

Although HADS-D showed no significant group-by-time interaction through postintervention (*F*_1.97, 184.92_=1.68, *P*=.096) or follow-up (*F*_2.79, 262.12_=1.56, *P*=.101), the treatment group demonstrated a notable reduction from baseline (mean 12.19, SD 4.10) to week 10 (mean 10.23, SD 4.63) compared to more modest changes in the control group from baseline (mean 11.00, SD 3.43) to week 10 (mean 10.25, SD 4.08), with a small effect size (Cohen d=0.30). The estimated mean change differences between groups for HADS-D were 0.77 (95% CI –0.96 to 2.5) at week 5, –0.02 (95% CI –1.81 to 1.77) at week 10, and –0.02 (95% CI –1.82 to 1.78) at week 15.

### Safety and Adherence

Safety and adherence analyses were conducted only for the treatment group. Throughout the study period, no severe adverse events were reported by any participants receiving Anzeilax. Adherence to Anzeilax was defined as completing at least 80% of the prescribed usage frequency (20 sessions at 5 weeks and 40 sessions at 10 weeks), as measured using the app usage log data. During the trial, 34 (70.8%) and 31 (64.6%) participants in the treatment group maintained at least 80% of the prescribed usage frequency at weeks 5 and 10, respectively.

## Discussion

### Principal Findings

This study demonstrated the superior efficacy of Anzeilax, a novel ACT-based DTx incorporating context-sensitive self-talk, when combined with TAU for the treatment of GAD. Significant between-group differences were observed in both FAS and PP analyses, with particularly strong outcomes in the PP analysis. The magnitude of treatment effects differed between the FAS (Cohen d=0.60) and PP (Cohen d=0.95) analyses. This discrepancy is methodologically consistent with the underlying principles of these analytical approaches [54,55]. FAS provides a conservative estimate of real-world effectiveness by including all randomized participants regardless of adherence, while PP measures efficacy under optimal adherence conditions. The higher effect size in PP analysis emphasizes the importance of treatment engagement in maximizing therapeutic outcomes.

Although the adjusted mean difference was slightly below the established GAD-7 minimal clinically important difference (MCID) of four points [56], the results indicated meaningful clinical improvement in anxiety symptoms. The standardized response mean (SRM), a commonly used effect size measure for quantifying the magnitude of clinical change and contributing to the estimation of the MCID, further supports the clinical relevance of these findings (see Section 11 in Multimedia Appendix 2) [56-58]. The interpretation is further supported by consistent positive outcomes across secondary measures.

The intervention’s effectiveness was demonstrated by improvements across multiple anxiety measures, notably a substantial reduction in BAI scores (Cohen d=0.50). The concurrent reduction in GAD-7 and BAI scores highlights a broad therapeutic impact, as these instruments assess complementary dimensions of anxiety. The GAD-7 focuses on cognitive and emotional symptoms, whereas the BAI captures physical manifestations [59]. This comprehensive improvement suggests potential applications beyond GAD to conditions with prominent physical symptoms, such as PD [60].

The significant improvement in PSWQ scores highlights the treatment’s efficacy in addressing pathological worry, a core feature of GAD. The PSWQ is specifically designed to distinguish pathological worry from normal anxiety by measuring the user’s excessive, pervasive, and uncontrollable nature [61]. Although the GAD-7 assesses broader anxiety symptoms, it offers a more nuanced evaluation of the cognitive worry component central to GAD. The synchronal improvements in both measures demonstrate the comprehensive therapeutic impact of Anzeilax on general anxiety symptoms and specific cognitive mechanisms underlying pathological worry.

Although past research on ACT manifested a positive expectation in reducing depressive symptoms [62,63], our results are inconsistent with those findings. Several factors may account for this outcome. First, the fully automated approach used in this study may have contributed to the discrepancy. In previous research, therapists were likely to actively involved in the treatment program [23,64], whereas we used a fully automated approach without human interaction. Given that therapeutic alliance and human contact are critical components in addressing depressive symptoms [65-67], this methodological difference may have influenced the results. Second, the inclusion of participants without comorbid MDD may have affected the findings. Specifically, 21 (43.8%) participants in the treatment group and 25 (52.1%) in the control group did not have MDD as a comorbid condition. This suggests that not all participants exhibited depressive symptoms substantial enough to contribute meaningfully to the analysis.

To further examine this possibility, subgroup analyses were conducted comparing participants with and without MDD. Independent-sample *t* tests revealed no significant differences in GAD symptom reduction between the two groups. In addition, linear regression analyses showed that MDD status is not a significant predictor of GAD change, indicating that the observed improvements in anxiety symptoms are not dependent on the presence or absence of comorbid depression. These results suggest that the presence of comorbid MDD does not significantly influence treatment response. Although this supports the robustness of the intervention across diagnostic subgroups, we recognize the clinical heterogeneity of the sample as a potential limitation. The absence of a significant modifying effect should be interpreted conservatively, as the sample size was not calculated to detect interaction effects across diagnostic groups (see Section 12 in Multimedia Appendix 2).

To evaluate the lasting effect of Anzeilax, follow-up assessments were conducted at 5 weeks postintervention. The therapeutic effects exhibited differential patterns across outcome measures. The treatment group maintained reductions in anxiety symptoms, as measured by the GAD-7 and BAI, whereas the PSWQ and HADS-A scores showed some rebound during the follow-up period. This divergence may reflect the distinct dimensions of anxiety assessed by each measure: the GAD-7 assesses core GAD symptoms, the BAI emphasizes physical manifestations of anxiety, HADS-A focuses on general emotional tension and fear, and the PSWQ evaluates worry persistence. The latter dimensions may be less stable due to the chronic and dynamic nature of these symptoms. Notably, these findings suggest that Anzeilax provides comprehensive effectiveness across various anxiety dimensions during active use, while maintaining particularly strong effects on core anxiety symptoms even after discontinuation.

The findings are consistent with Anzeilax’s therapeutic mechanism, which integrates ACT principles with context-sensitive self-talk to enhance psychological flexibility and modulate anxiety-related cognitive and behavioral processes within a contextual behavioral science framework [68]. Although Anzeilax is designed to improve individuals’ relationship with worry rather than eliminate it completely, participants may experience recurring worry loops when not actively engaging with the DTx. Although a 10-week intervention period appears sufficient to initiate cognitive shift through self-talk practices, more deeply ingrained worry habits may require extended or more intensive intervention to sustain consistent improvement during follow-up periods.

The positive outcomes observed in this study can be attributed to the innovative integration of self-talk within the ACT framework. By using context-sensitive self-talk strategies, Anzeilax appeared to enhance the effectiveness of cognitive defusion techniques central to ACT. The structured vocalization process during negative emotional states likely facilitates psychological distancing from anxious thoughts, whereas self-referencing techniques during positive states reinforce adaptive coping patterns. This dual approach, rooted in self-regulation theory and ACT principles, effectively addresses the challenges individuals with GAD face in emotional processing and regulation.

Additionally, the self-talk strategy encourages users to actively engage with the Anzeilax program by verbalizing its content aloud and listening to their own words [69,70]. This deliberate action not only reinforces the therapeutic concepts but also fosters deeper immersion in the intervention. By transforming passive engagement into active participation, this approach may significantly enhance the delivery and effectiveness of ACT’s core mechanisms, potentially contributing to therapeutic improvement in individuals with GAD.

Anzeilax is a noninvasive treatment method with minimal risk of adverse events or device-related effects. The absence of adverse events during our trial suggests that Anzeilax has a favorable safety profile when used as prescribed for GAD treatment. However, as a self-guided DTx, implementing a systematic reporting mechanism for technical issues will be essential when the product becomes available to the public.

### Clinical Implications

Our findings have several important clinical implications. The digital format of Anzeilax enhances accessibility, while sustained follow-up results indicate durable benefits for anxiety management. The integration of self-talk offers patients a feasible strategy for managing their symptoms, with observed improvements across anxiety-specific and general emotion regulation measures suggesting potentially broader benefits for overall emotional well-being.

### Limitations

This study had several limitations. First, the single-blind design (evaluator-blind) allowed participants to know their group assignments, potentially introducing response bias in self-reported outcomes. Although the sustained effects observed during the follow-up period suggest genuine therapeutic benefits rather than mere expectancy effects, future research should implement an active control condition using a sham device to minimize such bias. This approach would enable a more precise examination of the specific mechanisms underlying Anzeilax’s efficacy, independent of expectancy or placebo effects.

Second, although additional analyses were conducted to explore the role of comorbid depression, the study was not designed to detect interaction effects across clinical subgroups. The inclusion of participants with diverse diagnostic profiles introduces a level of heterogeneity that may limit the specificity and generalizability of the findings.

Given the recurring nature of psychological disorders, future research should investigate the sustainability of therapeutic benefits beyond the follow-up assessment at week 15. As shown in previous DTx studies, such as Carl et al [26], which included follow-up assessments at week 26 to evaluate treatment persistence, we recognize the importance of extended follow-up periods to better assess long-term effectiveness and sustainability. Additionally, to enhance understanding of the intervention’s MoA, subsequent studies should aim to distinguish the specific contributions of context-sensitive self-talk from standard ACT components.

Lastly, given the cultural context of the sample, the presence of culture-bound syndromes, such as *Hwabyung*, may have influenced symptom expression. Although this study did not include a specific assessment for *Hwabyung*, prior research has shown considerable symptomatic overlap between *Hwabyung* and GAD, particularly in emotional dysregulation, rumination, and affective tension [71,72]. These shared features correspond with cognitive and emotional processes targeted by ACT-based interventions. Thus, although cultural nuances were not directly captured, it is plausible that individuals with *Hwabyung*-like symptoms may have experienced therapeutic benefits through mechanisms, such as acceptance, defusion, and increased psychological flexibility. That being said, we acknowledge the need for greater diagnostic specificity in culturally embedded contexts. Future studies will incorporate validated *Hwabyung*-sensitive screening tools and examine outcomes stratified by symptom profile.

### Conclusion

In conclusion, this study provides evidence of the efficacy of Anzeilax and demonstrates that the integration of context-sensitive self-talk within an ACT-based DTx framework can effectively reduce anxiety symptoms in individuals with GAD. Consistent improvements across multiple measures of anxiety and worry, coupled with the maintenance of these gains at follow-up, suggest that this approach offers a promising scalable solution for the treatment of GAD. The absence of severe adverse events and reasonable adherence rates further support the feasibility and safety of implementing Anzeilax in clinical practice.

## Appendix

Multimedia Appendix 1 CONSORT-eHEALTH checklist (V 1.6.1).

Multimedia Appendix 2 Supplementary figures and tables.

## Abbreviations

ACT: acceptance and commitment therapy
BAI: Beck Anxiety Inventory
CBT: cognitive behavioral therapy
CONSORT: Consolidated Standards of Reporting Trials
DTx: digital therapeutic
FAS: full analysis set
GAD: generalized anxiety disorder
GAD-7: Generalized Anxiety Disorder 7-item scale
HADS: Hospital Anxiety and Depression Scale
HADS-A: Hospital Anxiety and Depression Scale – Anxiety
HADS-D: Hospital Anxiety and Depression Scale – Depression
IRB: Institutional Review Board
MCID: minimal clinically important difference
MDD: major depressive disorder
MoA: mechanism of action
PD: panic disorder
PP: per protocol
PSWQ: Penn State Worry Questionnaire
RCT: randomized controlled trial
SAD: social anxiety disorder
TAU: treatment as usual

## Data Availability

Data supporting the finding of this study are available from the corresponding author upon reasonable request.
