# Supplementary material for: Efficacy of a Smartphone-Based Digital Therapeutic (Anzeilax) in Generalized Anxiety Disorder: Randomized Controlled Trial
Source: J Med Internet Res. 2025 Oct 14;27:e69981. doi: 10.2196/69981 (PMC12569493; doi:10.2196/69981)
Supplement: Multimedia Appendix 2 [file jmir_v27i1e69981_app2.pdf]

**Multimedia Appendix 2.** Supplementary Materials for

# Efficacy of a Smartphone-Based Digital Therapeutic (Anzeilax) in Generalized Anxiety Disorder: Randomized Controlled Trial

Chanmi Park, MA; Hyunsil Song, BA; Hanna Kim, BA; Eunji Kim, MA; Hye-Jeong Jo, BA; Jee Hang Lee*, PhD; Jae-Jin Kim, MD, PhD; Jinwoo Kim*, PhD

*Corresponding author. E-mail: jinwoo@haii.io, jeehang@smu.ac.kr

## List of contents

1. *Anzeilax* DTx Application
2. Adjusted Mean Difference in GAD-7 (Week 10): Treatment vs. Control (PP)
3. Intervention Effects on Secondary Outcomes (PP)
4. Changes in Primary Outcomes and Secondary Outcomes (PP)
5. Primary and Secondary Outcome Measurements
6. The Process of Randomization
7. Missing Data Analysis and Multiple Imputation
8. Primary Analysis – Adjusted ANCOVA
9. Secondary Analysis – Linear Mixed-Effects Model (LMM)
10. Sensitivity Analysis
11. Standardized Response Mean (SRM)
12. Impact of Comorbid MDD on GAD Treatment Response

## Supplementary Figures

**Figure S1.** A snapshot of *Anzeilax*
**Figure S2.** Comparison of the adjusted mean difference (week 10) in GAD-7 between treatment and control groups in PP
**Figure S3.** Changes in primary outcome (GAD-7) and secondary outcome (BAI, PSWQ, HADS-A, HADS-D); PP
**Figure S4.** Primary Endpoint – Change in GAD-7 between baseline and post-treatment (FAS)
**Figure S5.** Change in measurement scores over time (LMM) (FAS)
**Figure S6.** Forest plot of Cohen *d* (FAS)

# Supplementary Table

**Table S1.** Impact of the intervention on the secondary outcome measures (PP)
**Table S2.** Little's MCAR Test Results
**Table S3.** Adjusted ANCOVA Results (Pooled) for GAD-7
**Table S4.** Fixed effects estimates from the pooled Linear Mixed-Effects Model
**Table S5.** Pooled random effects variance and intraclass correlations
**Table S6.** Dropout Patterns
**Table S7.** Sensitivity analysis results (Time*Group results)
**Table S8.** Pre-post treatment outcomes for *Anzeilax* for SRM calculation

## 1. *Anzeilax* DTx Application

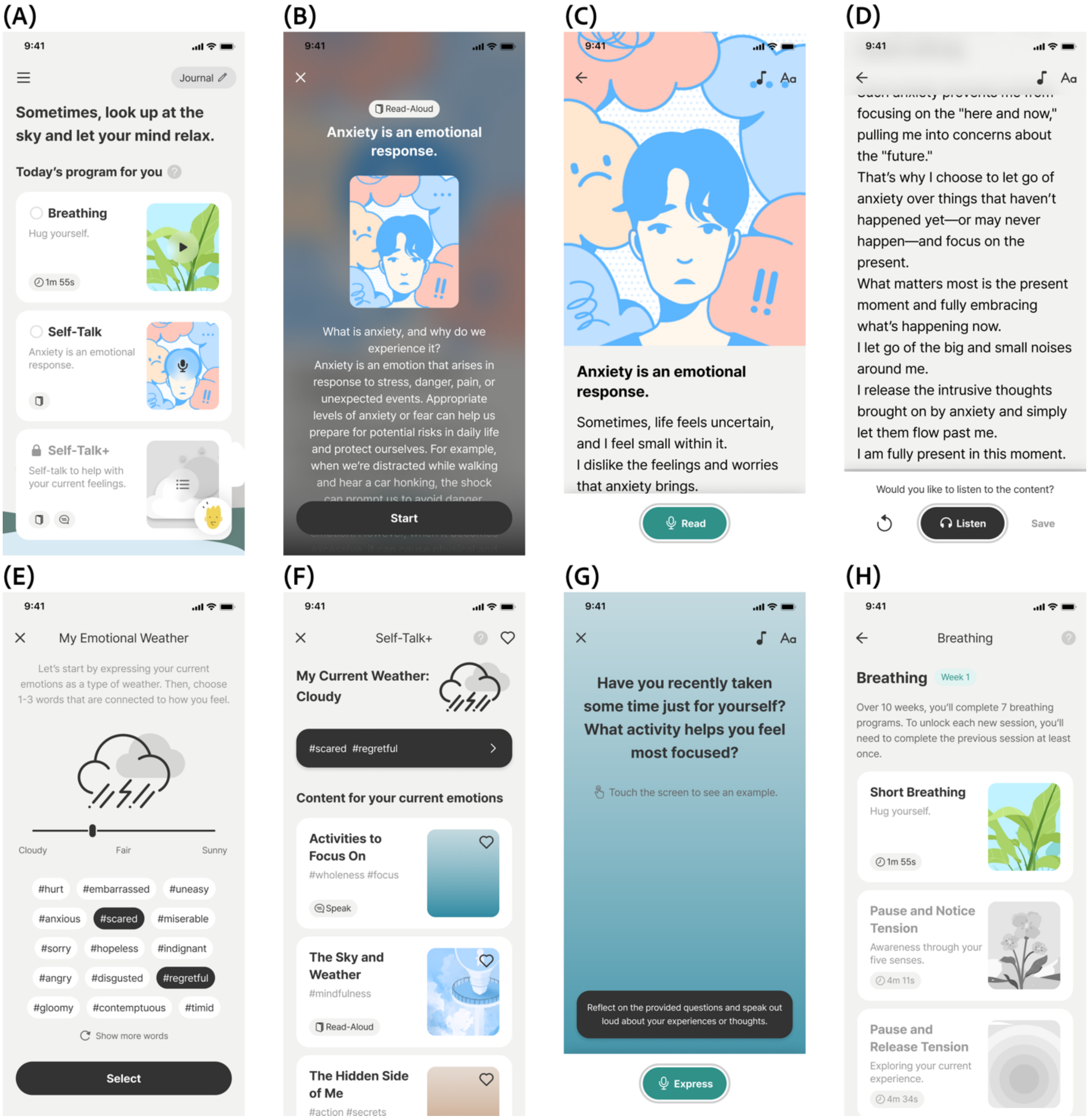


**Figure S1. A snapshot of *Anzeilax*.** An application interface showing the main access page for breathing, self-talk, and self-talk plus programs. It also illustrates key components: (A) *Anzeilax* home. (B) Introduction session of self-talk content. (C–D) Self-talk program interfaces before recording (C) and listening phases (D). (E–G) Self-talk plus features including emotion selection (E), content list (F), and self-verbalization session (G). (H) breathing program selection menu.

## 2. Adjusted Mean Difference in GAD-7 (Week 10): Treatment vs. Control (PP)

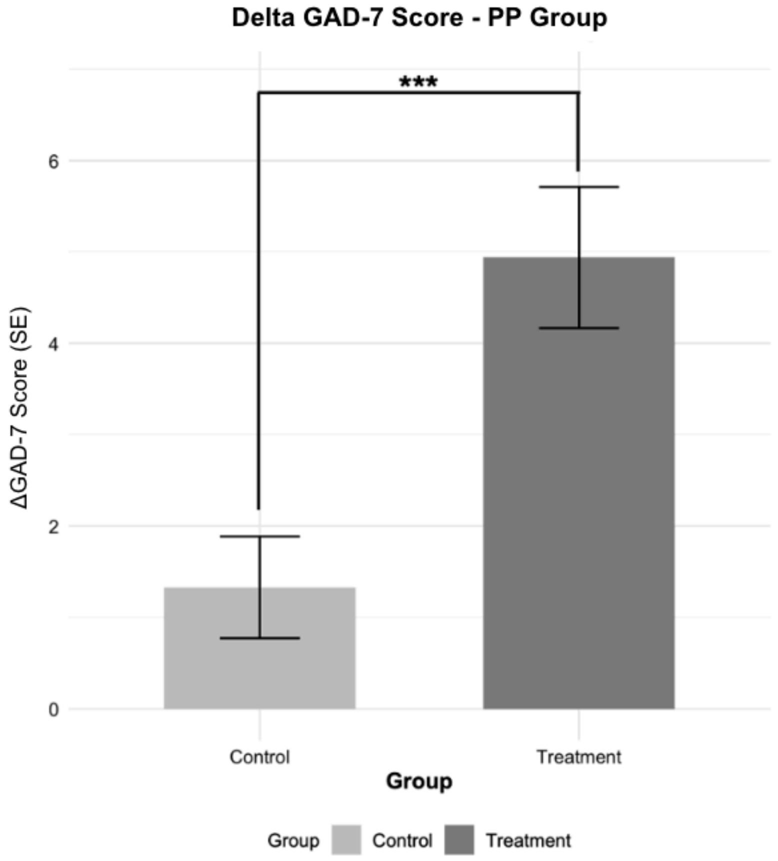


**Figure S2.** Comparison of the adjusted mean difference (week 10) in GAD-7 between treatment and control groups in PP.

## 3. Intervention Effects on Secondary Outcomes (PP)

**Table S1.** Impact of the intervention on the secondary outcome measures (PP)

| | Treatment Mean (SD) | Control Mean (SD) | Estimate of mean change difference (95% CI) | *P* value (Group x time) | Effect size (Cohen *d*) |
|---|---|---|---|---|---|
| **GAD-7**[a] | | | | | |
| Baseline | 12.1 (3.57) | 12.4 (3.45) | -0.27 (-1.93, 1.38) | <.001 | 0.92 |
| Midintervention (Week 5) | 8.61 (3.78) | 10.3 (3.89) | -1.67 (-3.45, 0.12) | | |
| Postintervention (Week 10) | 7.16 (3.99) | 11.0 (4.29) | -3.88 (-5.69, -2.07) | | |
| Follow-up (Week 15) | 6.77 (3.83) | 10.2 (4.76) | -3.4 (-5.2, -1.6) | <.001 | N/A |
| **BAI**[b] | | | | | |
| Baseline | 25.0 (11.4) | 25.3 (8.54) | -0.23 (-5.45, 4.99) | <.001 | 0.79 |
| Midintervention (Week 5) | 20.2 (11.0) | 23.6 (10.8) | -3.39 (-8.9, 2.11) | | |
| Postintervention (Week 10) | 15.0 (10.6) | 23.2 (9.99) | -8.21 (-13.1, -3.28) | | |
| Follow-up (Week 15) | 14.7 (8.93) | 23.2 (10.9) | -8.48 (-12.8, -4.13) | <.001 | N/A |
| **PSWQ**[c] | | | | | |
| Baseline | 57.2 (4.64) | 55.7 (4.68) | 1.48 (-0.8, 3.75) | <.001 | 0.92 |
| Midintervention (Week 5) | 53.6 (4.94) | 56.2 (5.06) | -2.59 (-4.98, -0.21) | | |
| Postintervention (Week 10) | 52.9 (4.84) | 55.8 (4.93) | -2.9 (-5.15, -0.65) | | |
| Follow-up (Week 15) | 54.0 (4.94) | 55.1 (7.31) | -1.08 (-3.98, 1.83) | 0.002 | N/A |
| **HADS-A**[d] | | | | | |
| Baseline | 13.7 (3.67) | 13.2 (2.63) | 0.52 (-1.09, 2.13) | <.001 | 0.89 |
| Midintervention (Week 5) | 11.6 (3.75) | 12.8 (3.47) | -1.23 (-3.02, 0.55) | | |
| Post-intervention | 9.61 (3.85) | 12.2 (3.82) | -2.54 (- | | |

| | | | | | |
|---|---|---|---|---|---|
| (Week 10) | | | 4.33, -0.75) | | |
| Follow-up (Week 15) | 10.4 (4.18) | 12.2 (4.34) | -1.78 (-3.86, 0.31) | 0.002 | N/A |
| **HADS-D**[e] | | | | | |
| Baseline | 12.0 (4.10) | 11.0 (3.43) | 0.97 (-0.97, 2.91) | 0.017 | 0.55 |
| Midintervention (Week 5) | 10.4 (4.65) | 10.3 (3.73) | 0.05 (-2.08, 2.18) | | |
| Postintervention (Week 10) | 9.0 (4.63) | 10.2 (4.08) | -1.22 (-3.31, 0.88) | | |
| Follow-up (Week 15) | 9.29 (4.54) | 10.4 (4.21) | -1.12 (-3.22, 0.97) | 0.017 | N/A |

[a] Generalized Anxiety Disorder 7-item scale.

[b] Beck Anxiety Inventory.

[c] Penn State Worry Questionnaire.

[d] Hospital Anxiety and Depression Scale-Anxiety.

[e] Hospital Anxiety and Depression Scale- Depression.

## 4. Changes in Primary Outcomes and Secondary Outcomes (PP)

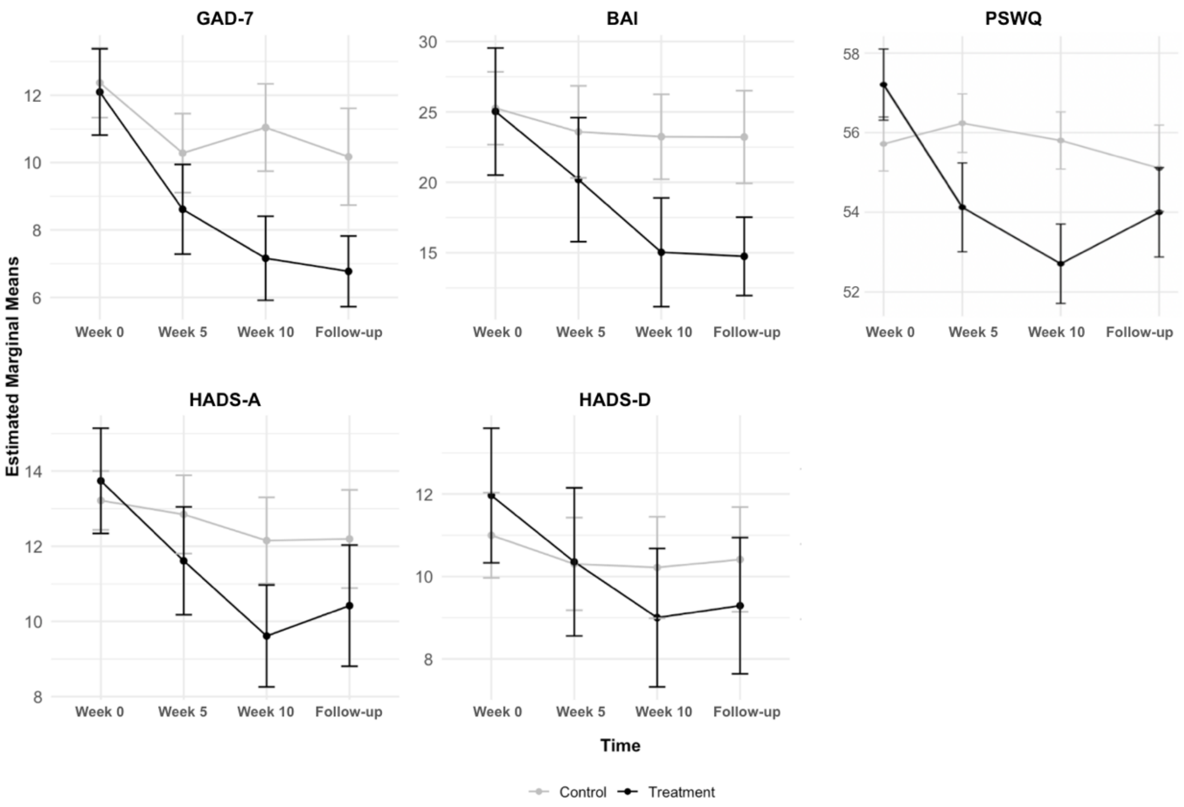


**Figure S3. Changes in primary outcome (GAD-7) and secondary outcome (BAI, PSWQ, HADS-A, HADS-D); PP.** At each plot, X-axis refers to a week (week 0, 5, 10 and follow-up), and Y-axis refers to a score of each outcome (estimated marginal mean).

## 5. Primary and Secondary Outcome Measurements

Assessments were conducted at four time points: baseline (week 0), mid-intervention (week 5), post-intervention (week 10), and follow-up (week 15). Baseline measurements were completed in person at the hospital, while subsequent assessments were conducted online. The primary outcome measure was Generalized Anxiety Disorder 7-item (GAD-7) scale, a validated tool for measuring the severity of generalized anxiety symptoms (Cronbach α=.92) (Spitzer et al., 2006). The GAD-7 consists of seven items rated on a 4-point Likert scale (0–3), with a total score range of 0–21. Based on the score, anxiety severity is classified as minimal (0–4), mild (5–9), moderate (10–14), or severe (15–21). In this study, a GAD-7 score of 10 or higher was used as the cut-off for determining clinically significant anxiety.

Secondary outcomes included specific domains of anxiety, evaluated using standardized measures. Cognitive and physical symptoms of anxiety were assessed with the Beck Anxiety Inventory (BAI), which consists of 21 items rated on a 4-point Likert scale, with a total score range of 0–63 (Cronbach α=.92) (Beck et al., 1988). Anxiety severity is categorized as minimal (0–7), mild (8–15), moderate (16–25), or severe (26–63). Worry severity was measured using the Penn State Worry Questionnaire (PSWQ), a 16-item scale rated on a 5-point Likert scale, with a total score range of 16–80 (Cronbach α=.86-.91) (Dear et al., 2011). Based on the score, individuals are classified as not anxious or a worrier (≤29), bothered by worries but below the clinical range (30–52), experiencing some problems with worry and potentially benefiting from treatment (52–65), or a chronic worrier in need of treatment (≥66). Overall anxiety and depression symptoms were evaluated using the Hospital Anxiety and Depression Scale (HADS), which consists of 14 items rated on a 4-point Likert scale, with separate subscales for anxiety (HADS-A) (Cronbach α=.68-.93) and depression (HADS-D) (Cronbach α=.67-.90), each ranging from 0 to 21. Scores are categorized as normal (0–7), borderline abnormal (8–10), or abnormal (11–21) (Bjelland et al., 2002).

## 6. The Process of Randomization

Before the trial began, the randomization officer generated the randomization numbers, including envelope numbers, and provided them to the researchers in sealed, opaque envelopes to ensure allocation concealment.

A face-to-face baseline assessment was conducted at Gangnam Severance Hospital, where participants underwent initial evaluations. Participants who met the eligibility criteria and provided informed consent were assigned a randomization number in the order of their enrollment. To maintain blinding and ensure the integrity of the randomization process, the sealed envelopes were opened by an individual independent of participant enrollment. Each participant then drew a slip marked "treatment group" or "control group" under the supervision of the Clinical Research Coordinator (CRC), who documented the randomization date and signed the slip. The time of participant registration and the name of the person opening the envelope were recorded for documentation purposes. Based on the assigned number, participants were allocated to either the digital therapeutic device + treatment as usual (TAU) group or the TAU-only control group.

Additionally, at the end of the trial, the researchers conducted a verification process to confirm that the serial numbers of the opened envelopes matched the records of enrolled participants, and the remaining sealed envelopes were cross-checked to confirm procedural accuracy.

As this study was evaluator-blinded rather than investigator-blinded, the randomization records were managed by the principal investigator and the designated randomization officer at the trial site. The randomization number, along with the participant's screening number, served as the identification code throughout the study period.

## 7. Data Missing Analysis and Multiple Imputation

### (1) Data Missing Analysis

The dataset analyzed included scores of GAD-7, BAI, PSWQ, and HADS(HADS-D, HADS-A) measured over four time points (week 0 – baseline, week 5, week 10 – end of intervention, and week 15).

Prior to analysis, missing data were assessed to determine the most appropriate imputation method. Little's Missing Completely At Random (MCAR) Test was conducted on each metric, using the mcar_test() function from the mice package (v.3.16.0) in R.

Results indicated that all variables exhibited non-significant p-values, suggesting that missing data were consistent with the MCAR assumption. Specifically, chi-square statistics ranged from $\chi^2$=1.81, p=.937 (GAD-7) to $\chi^2$=10.7, p=.0981 (HADS-A), suggesting that missing values do not systematically differ in variability between observed and missing cases (Table S2).

Further supporting this, participant log documenting dropout time points – most of which were during the first or second weeks of the study – and self-reported reasons for discontinuation citing "busy schedule" as the primary reason for dropping out suggests that missingness is likely related to external factors rather than the underlying variables of interest. Based on these findings, Multiple Imputation was deemed to be suitable for handling the missing data.

**Table S2.** Little's MCAR Test Results.

| Measure | $\chi^2$ (df = 6) | p-value |
|---|---|---|
| GAD-7 | 1.81 | 0.937 |
| BAI | 7.82 | 0.251 |
| PSWQ | 5.34 | 0.501 |
| HADS-A | 10.7 | 0.0981 |
| HADS-D | 7.65 | 0.265 |

### (2) Multiple Imputation

Having confirmed the randomness of the missing data patterns, multiple imputation by chained equation (MICE) was used to handle missingness, via the mice() package in R (method = "21.pan" to account for variability within patients), utilizing the linear mixed-effects model-based imputation model to account for the random effects that could occur within individuals over repeated measures. Given the sample size that we had, and the fact that the missing data proportion was 0.15, we deemed 20 imputation sets to be suffice(m = 20) (MCCB, 2014; Graham et al., 2007) and each imputation dataset ran for 10 cycles to ensure stable estimates(m = 20, maxit = 10). Imputation was performed separately for each metric to maintain imputation consistency across all metrics. Convergence of imputations was observed via trace plots and density plots, confirming that the imputed data reflected the observed data distributions.

## 8. Primary Analysis – Adjusted ANCOVA

To study the treatment effect on the treatment and control groups between baseline and the final treatment time point (Week 10), an ANCOVA model was fitted to examine the effect of the Treatment group on Week 10 scores, while adjusting for the baseline GAD-7 scores. The ANCOVA model results for the 20 imputations were pooled following Rubin's rule (Rubin, 1987), presented in Table S3.

**Table S3.** Adjusted ANCOVA Results (Pooled) for GAD-7.

| | Estimate (β) (CI) | SE | t-value | p-value | $\eta^2_{partial}$ | Cohen *d* |
|---|---|---|---|---|---|---|
| Intercept | 5.00 (2.00, 7.99) | 1.53 | 3.27 | 0.00112** | 0.0135 | 3.27 |
| Group | -3.13 (-4.72, -1.55) | 0.807 | -3.88 | 0.000114*** | 0.0223 | -3.88 |
| Baseline (Week 0) | 0.487 (0.259, 0.715) | 0.116 | 4.19 | 0.0000318*** | 0.0262 | 4.19 |

*Pooled adjusted $R^2$: 0.281

The treatment group demonstrated a statistically significant reduction in GAD scores at Week 10 compared to the control group ($P < .001$). The effect size (Cohen *d* = -3.88) suggests strong practical effect of the treatment. The adjusted $R^2$ of the ANCOVA model, pooled across imputations, was calculated as 0.281, indicating that the model explains 28.1% of the variance in post-treatment GAD score was explained by the model.

To further explore the treatment effect of *Anzeilax* over time while capturing the within-subject variability and other random effects, we conducted a linear mixed-effects model (LMM) to examine the treatment effect across multiple time points (week 0, week 5, week 10, week 15 follow-up) and between groups.

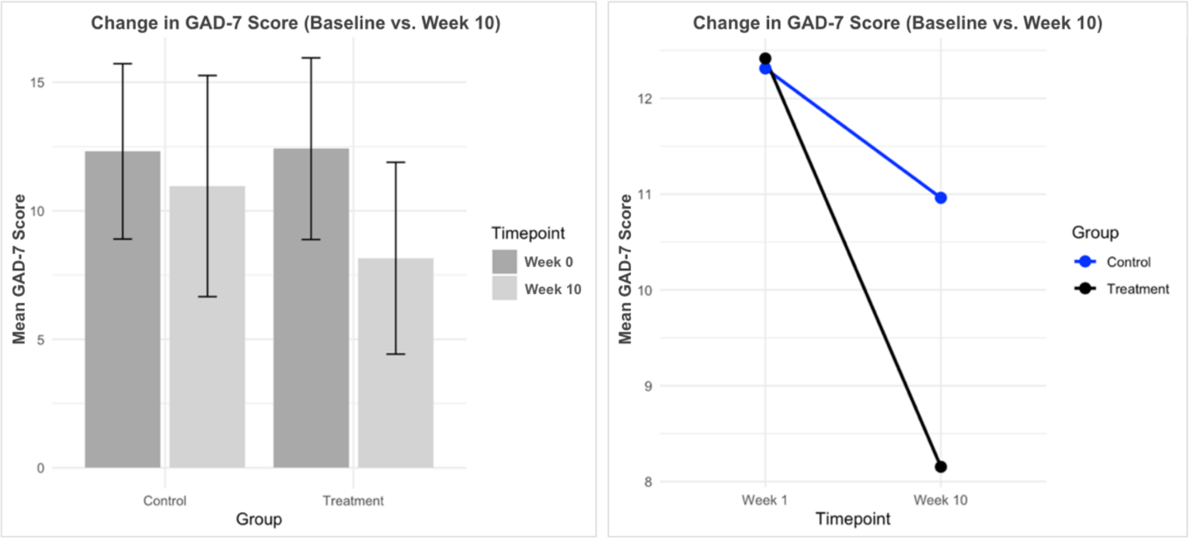


**Figure S4.** Primary Endpoint – Change in GAD-7 between baseline and post-treatment (FAS).

## 9. Secondary Analysis – Linear Mixed-Effects Model (LMM)

There are mixed opinions on the question of whether missing data aimed for linear mixed-effects model require imputation, given that LMMs are often known to handle missing data under the MAR assumption (Gabrio et al, 2022; West et al, 2012). However, having conducted LMM analysis for both imputed and unimputed data, although the statistical significance results were similar, the imputed data provided more conservative p-values with higher degrees of freedom. This suggests that the imputed data produced p-values with higher degrees of freedom, indicating improved precision in the estimates.

Thus, the linear mixed-effects model(LMM) was fitted to each of the 20 imputed dataset and pooled using the Rubin's rules(1987), accounting for both within-imputation variance and between-imputation variance to ensure valid standard errors and confidence intervals (Table S4). The model included a random intercept for each participant to account for individual variability, and incorporated an autoregressive correlation structure of order 1 (corAR1) to model within-subject correlations over time. The model is specified as:

$$S_{it} = \beta_0 + \beta_1 G_i + \beta_2 T_t + \beta_3 (G_i . T_t) + r_i + \varepsilon_{it}$$

Where:

- $S_{it}$: Score for participant i at time t
- $G_i$: Treatment group assignment (Control = 0, Treatment = 1)
- $T_t$: Time point t (t=Week 0(baseline), 5, 10, 15(follow-up))
- $r_i$: random intercept for each participant to capture individual variability
- $\varepsilon_{it} \sim N(0, \sigma^2)$*:* residual error term

To account for multiple comparisons, a Bonferroni correction was applied to the interaction terms in the LMM analysis to control the family-wise error rate. Pooled estimates provided fixed effects for treatment, time, and their interaction presented in Table S4. Moreover, Cohen *d* was computed to quantify the magnitude of the treatment effect at each time point, based on the estimated interaction terms in the LMM.

### Results

### (1) Main effects:

There were no significant baseline differences between the treatment and control groups across all outcome measures, confirming comparability between groups at Week 0. For the GAD-7 measure, the difference between groups approached significance by Week 5 and became significant by Week 10 (*P* = .002, Bonferroni *P* = .019), with this effect remaining significant at follow-up. Similarly, for the BAI measure, no significant effects were observed until Week 10, when a significant treatment effect emerged (*P* = .004, Bonferroni *P* = .033), which persisted at follow-up.

For the PSWQ measure, treatment effects were observed earlier, with significant differences appearing from Week 5 onwards (*P* = .003, Bonferroni *P* = .022). In contrast, for the HADS-D

measure, treatment effects were not observed until Week 10, with a significant effect persisting at follow-up but losing significance after Bonferroni correction. The HADS-A measure followed a similar pattern, with a trend toward significance at Week 5, followed by a significant effect at Week 10 ($P$ = .006, Bonferroni $P$ = .049). While this effect persisted at follow-up, it did not maintain significance after correction for multiple comparisons.

Overall, these results suggest that treatment effects emerged gradually after Week 5, with the most consistent and sustained improvements observed in the GAD-7, BAI, and PSWQ measures. The HADS metrics demonstrated later-emerging treatment effects, with some effects diminishing after adjustment for multiple comparisons.

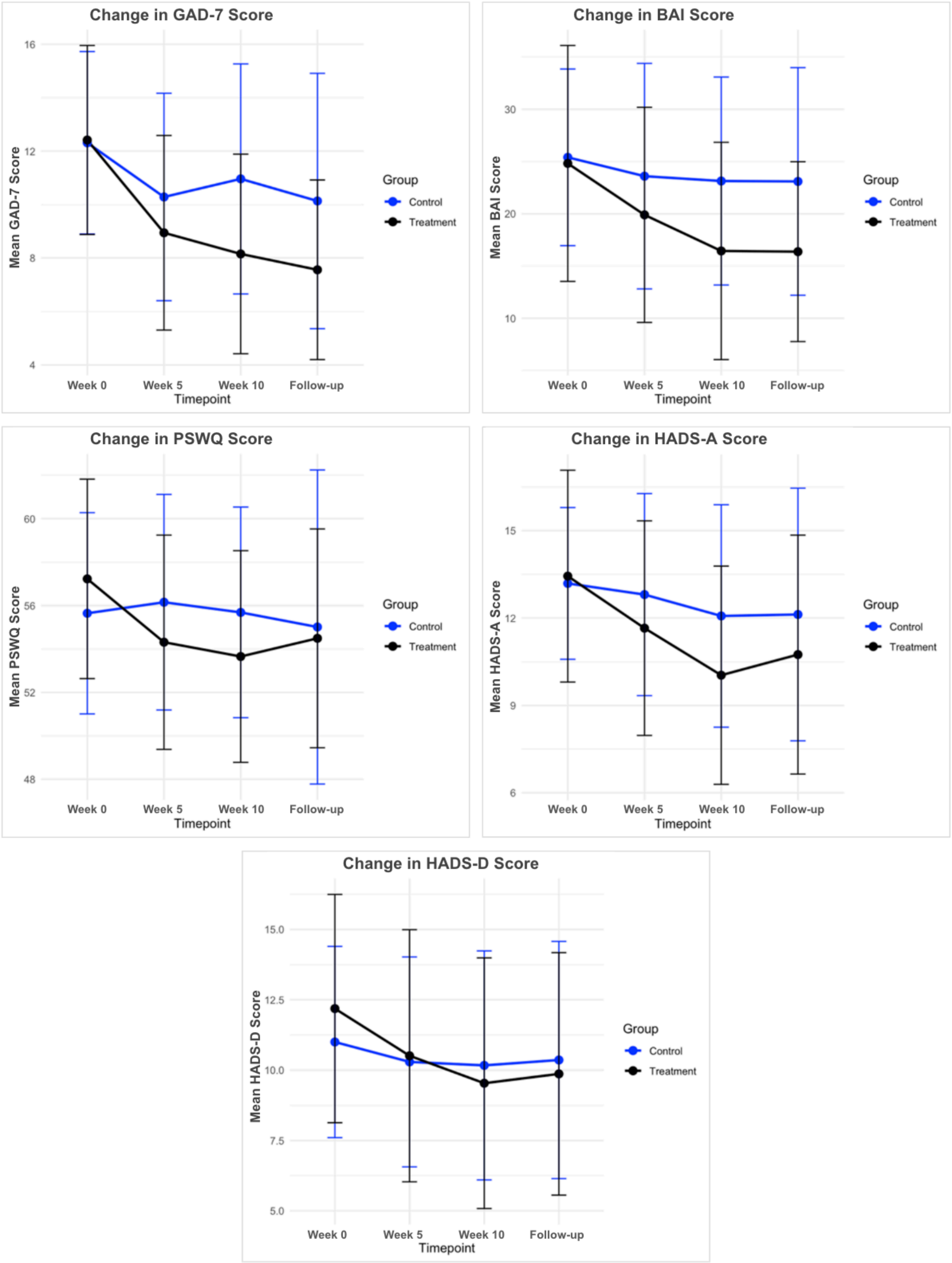


**Figure S5.** Change in measurement scores over time (LMM) (FAS).

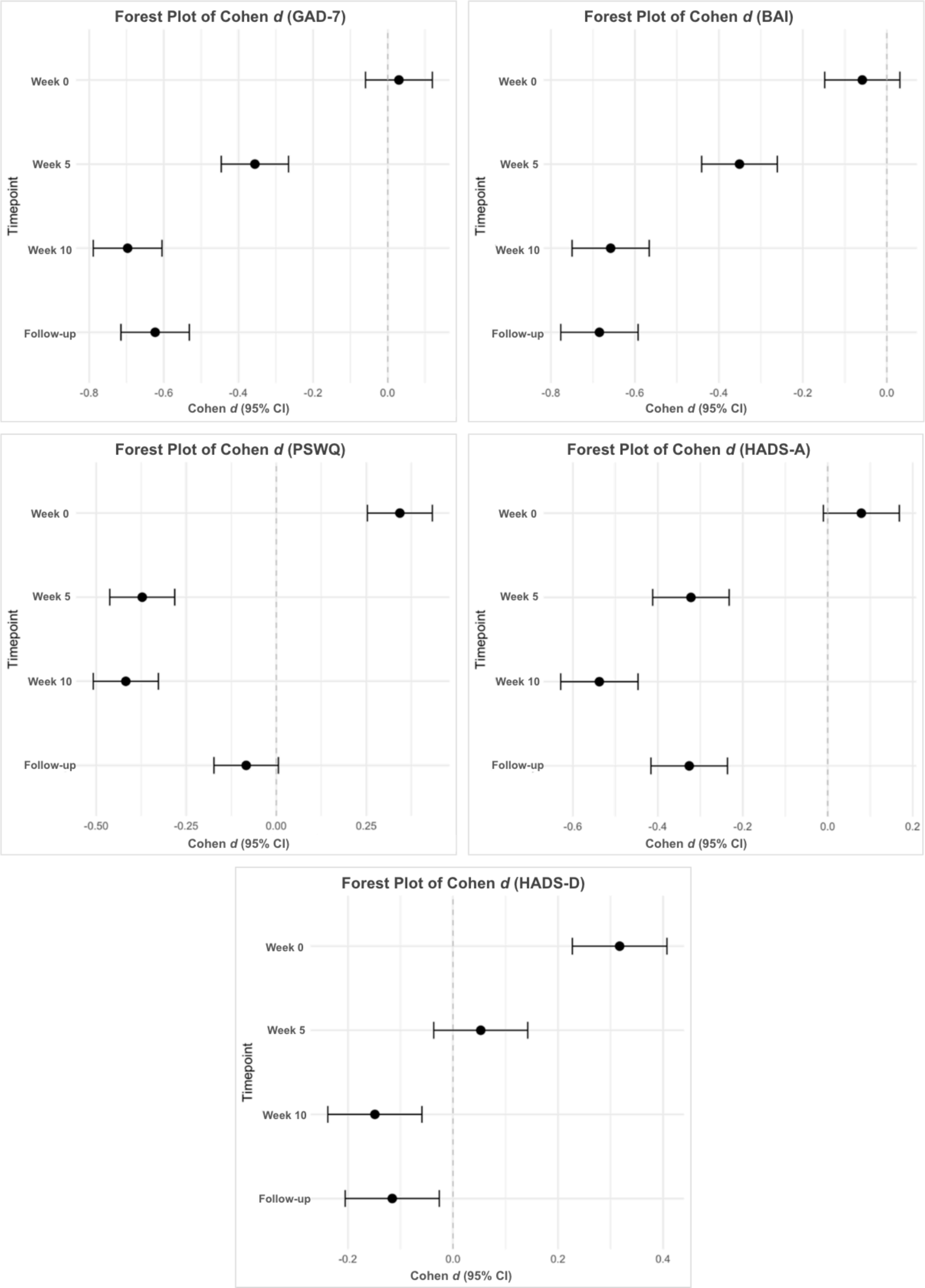


**Figure S6.** Forest plot of Cohen *d* (FAS).

### (2) Fixed Effects Estimates

The LMM analysis revealed significant time-by-group interactions for all primary and secondary outcome measures. In GAD-7, significant group differences emerged from Week 5 (β=-1.74, 95% CI: -0.82, -0.0015, *P*=.00492) and became more pronounced at Week 10 (β=-3.21, 95% CI: -1.17, -0.34, *P*=.0004) and Follow-up (β=-2.74, 95% CI: -1.06, -0.23, *P*=.0022). The treatment effect sizes progressively increased from Week 5 (Cohen *d*=-0.41) to Week 10 (Cohen *d*=-0.76) and slightly decreased at Follow-up (Cohen *d*=-0.65).

BAI scores showed a similar pattern with significant group differences appearing at Week 10 (β=-6.52, 95% CI: -1.02, -0.20, *P*=.0034) and strengthening at Follow-up (β=-6.73, 95% CI: -1.00, -0.26, *P*=.0009), with moderate effect sizes (Week 10: Cohen *d*=-0.61; Follow-up: Cohen *d*=-0.63).

PSWQ demonstrated significant improvements starting from Week 5 (β=-3.63, 95% CI: -1.11, -0.26, *P*=.0018) and maintained through Week 10 (β=-3.68, 95% CI: -1.17, -0.21, *P*=.0046), with large effect sizes (Week 5: Cohen *d*=-0.68; Week 10 Cohen *d*=-0.69).

For depression measures, HADS-D showed significant improvement at Week 10 (β=-1.87, 95% CI: -0.83, -0.06, *P*=.0231) and Follow-up (β=-1.85, 95% CI: -0.81, -0.07, *P*=.0194), with moderate effect sizes (Cohen *d*=-0.45 and Cohen *d*=-0.44, respectively). Similarly, HADS-A demonstrated significant reduction at Week 10 (β=-2.37, 95% CI:-1.05, -0.21, *P*=.0031) and Follow-up (β=-1.78, 95% CI: -0.86, -0.06, *P*=.023), with moderate to large effect sizes (Cohen *d*=-0.61 and Cohen *d*=-0.46, respectively).

These results consistently demonstrate that the treatment group showed superior improvements across all anxiety and depression measures compared to the control group, with effects generally emerging by Week 5 or 10 and maintaining through the follow-up period.

**Table S4.** Fixed effects estimates from the pooled Linear Mixed-Effects Model.

| Timepoint | Treatment Mean (SD) | Control Mean (SD) | Estimate of Mean Change (β) (95% CI) | df | p-value (Group × Time) | Bonferroni-adjusted p-value | Cohen *d* (SE) |
|---|---|---|---|---|---|---|---|
| **GAD** | | | | | | | |
| Week0 (Baseline) | 12.42 (3.54) | 12.31 (3.41) | 0.10 (-1.46, 1.67) | 279.993 | 0.896 | 1.000 | 0.03 (0.0456) |
| Week 5 | 8.94 (3.64) | 10.28 (3.88) | -1.44 (-3.20, 0.32) | 169.956 | 0.107 | 0.857 | -0.36 (0.0460) |
| Week 10 | 8.15 (3.73) | 10.96 (4.30) | -2.91 (-4.77, -1.05) | 159.897 | 0.002** | 0.019* | -0.70 (0.047) |
| Follow-up | 7.56 (3.36) | 10.13 (4.78) | -2.68 (-4.44, -0.92) | 228.200 | 0.003** | 0.024* | -0.62 (0.0467) |
| **BAI** | | | | | | | |

| | | | | | | | |
|---|---|---|---|---|---|---|---|
| Week 0 (Baseline) | 24.81 (11.29) | 25.40 (8.45) | -0.58 (-4.69, 3.52) | 279.993 | 0.78 | 1.000 | -0.06 (0.0457) |
| Week 5 | 19.89 (10.29) | 23.59 (10.79) | -3.12 (-6.85, 0.62) | 258.935 | 0.101 | 0.810 | -0.35 (0.0460) |
| Week 10 | 16.44 (10.40) | 23.13 (9.95) | -6.11 (-10.26, -1.96) | 188.732 | 0.004** | 0.033* | -0.66 (0.0469) |
| Follow-up | 16.37 (8.61) | 23.09 (10.89) | -6.14 (-10.31, -1.96) | 189.396 | 0.004** | 0.033* | -0.68 (0.0470) |
| **PSWQ** | | | | | | | |
| Week 0 (Baseline) | 57.23 (4.59) | 55.65 (4.63) | 1.58 (-0.53, 3.69) | 279.993 | 0.141 | 1.000 | 0.34 (0.0460) |
| Week 5 | 54.31 (4.93) | 56.15 (4.96) | -3.42 (-5.65, -1.19) | 263.291 | 0.003** | 0.022* | -0.37 (0.0460) |
| Week 10 | 53.66 (4.87) | 55.69 (4.85) | -3.61 (-6.01, -1.21) | 194.71 | 0.003** | 0.027* | -0.42 (0.0461) |
| Follow-up | 54.49 (5.03) | 55.01 (7.23) | -2.10 (-4.51, 0.30) | 192.879 | 0.086 | 0.691 | -0.08 (0.0457) |
| **HADS-A** | | | | | | | |
| Week 0 (Baseline) | 13.44 (3.64) | 13.19 (2.60) | 0.25 (-1.25, 1.75) | 279.993 | 0.744 | 1.000 | 0.08 (0.0457) |
| Week 5 | 11.65 (3.68) | 12.80 (3.47) | -1.40 (-2.81, 0.00) | 258.739 | 0.051 | 0.405 | -0.32 (0.0459) |
| Week 10 | 10.04 (3.75) | 12.07 (3.82) | -2.28 (-3.91, -0.66) | 194.23 | 0.006** | 0.049* | -0.54 (0.0465) |
| Follow-up | 10.74 (4.10) | 12.12 (4.33) | -1.63 (-3.27, 0.02) | 198.651 | 0.053* | 0.423 | -0.33 (0.0459) |
| **HADS-D** | | | | | | | |
| Week 0 (Baseline) | 12.19 (4.06) | 11.00 (3.40) | 1.19 (-0.47, 2.85) | 279.993 | 0.161 | 1.000 | 0.32 (0.0459) |
| Week 5 | 10.51 (4.48) | 10.29 (3.73) | -0.97 (-2.40, 0.46) | 259.464 | 0.182 | 1.000 | -0.05 (0.0457) |
| Week 10 | 9.53 (4.45) | 10.17 (4.07) | -1.82 (-3.43, -0.22) | 200.382 | 0.026* | 0.211 | -0.15 (0.0457) |
| Follow-up | 9.87 (4.31) | 10.36 (4.22) | -1.68 (-3.32, -0.04) | 190.666 | 0.044* | 0.353 | -0.12 (0.0457) |

### (3) Random Effects Estimates

Random effects estimates were calculated across 20 imputed datasets and pooled according to Rubin's Rules, the results presented in a summary table (Table S5). The intraclass correlation coefficient (ICC) was calculated to assess the proportion of variance attributable to between-subject variability.

Results revealed substantial between-subject variability across all five measures. ICC values ranged from 0.43 (GAD-7) to 0.59 (HADS-D), which indicates that 43 – 59% of the total variance

observed is due to between-subject differences. The mean patient-level variance and residual variance were all relatively similar suggesting that individual differences and within-person variabilities are similar across all metrics – which is well reflected in the ICC values.

**Table S5.** Pooled random effects variance and intraclass correlations.

| | **Variance (SE)** | **95% CI** | **ICC** |
|---|---|---|---|
| **GAD-7** | | | |
| PatientID | 6.57 (0.41) | ( 5.77 - 7.37 ) | 0.43 |
| Residual | 8.88 (0.39) | ( 8.11 - 9.65 ) | |
| **BAI** | | | |
| PatientID | 66.70 (2.91) | (61.00 – 72.41) | 0.56 |
| Residual | 52.05 (2.45) | ( 47.24 - 56.85 ) | |
| **PSWQ** | | | |
| PatientID | 12.86 (0.81) | (11.27 – 14.45 ) | 0.45 |
| Residual | 15.68 (0.55) | (14.60 – 16.75) | |
| **HADS-D** | | | |
| PatientID | 10.24 (0.49) | ( 9.28 – 11.20 ) | 0.59 |
| Residual | 7.21 (0.32) | ( 6.58 - 7.83 ) | |
| **HADS-A** | | | |
| PatientID | 6.88 (0.47) | ( 5.95 – 7.80 ) | 0.48 |
| Residual | 7.48 (0.37) | ( 6.74 - 8.21 ) | |

## 10. Sensitivity Analysis

To confirm the robustness of the analysis, several sensitivity analyses were conducted and although MAR is assumed, we have included models that explore the influence of the missingness of data on the outcome variable to confirm that the data analysis is robust to MNAR. The analyses conducted are as follows:

1. **Model 1: Predictive Mean Matching (PMM) Imputation with ANCOVA & LMM**
   To confirm that the results were not biased by the imputation method used, we have applied an alternative multiple imputation method using predictive mean matching (PMM), with the imputation model that includes PatientID as the grouping variable to account for individual differences over repeated measures – in addition to the existing time and group variables. The imputed datasets were analyzed using LMM (secondary analysis), keeping the model specifications identical to the primary approach. The only difference was the method used for multiple imputation(PMM instead of the two-level pan model). The results from both imputation methods were consistent.

2. **Model 2: LMM with 'time' as a random effect**
   To account for the possibility that the treatment effect may vary over time – meaning that each patient could experience the treatment effect at different time points - we have added time as a random effect in the LMM model. The multiple imputation method and other model variables remained unchanged. The results were consistent with those of the primary approach.

3. **Model 3: LMM Without Multiple Imputation**
   To assess the impact of missing data handling, we conducted a linear mixed-effects model without multiple imputation, using only the available data. The results remained similar to that of the primary approach, which suggests that the missing data handling did not bias the findings.

4. **Model 4: Pattern-Mixture Model (PMM)**
   A pattern-mixture model was fitted to explore potential bias due to missing data patterns. Participants were categorized into:

   a. Completers (participants with no missing data)
   b. Dropouts (participants who stopped participation entirely at some point)
   c. Intermittent missing (participants with some missing data but did not drop out completely)

The dropout patterns are summarized as in Table S6, which shows that the majority of participants were Completers (~78%), a smaller proportion were identified as Dropouts (~20%), and a small group (~2%) were categorized as having Intermittent Missing data.

**Table S6.** Dropout Patterns.

| Pattern | n | % |
|---|---|---|
| **GAD-7** | | |
| Completer | 75 | 78.1 |

| | | |
|---|---|---|
| Dropout | 19 | 19.8 |
| Intermittent Missing | 2 | 2.1 |
| **BAI** | | |
| Completer | 75 | 78.1 |
| Dropout | 19 | 19.8 |
| Intermittent Missing | 2 | 2.1 |
| **PSWQ** | | |
| Completer | 75 | 78.1 |
| Dropout | 19 | 19.8 |
| Intermittent Missing | 2 | 2.1 |
| **HADS-A** | | |
| Completer | 75 | 78.1 |
| Dropout | 19 | 19.8 |
| Intermittent Missing | 2 | 2.1 |
| **HADS-D** | | |
| Completer | 75 | 78.1 |
| Dropout | 19 | 19.8 |
| Intermittent Missing | 2 | 2.1 |

In the model, the Completers group was set as the reference group for the pattern variable, which allowed the estimated effects for the Dropout and Intermittent missing groups to assess their deviation from the Completers group, and thus, the impact of missingness on the output variable.

A mixed-effects model was applied, incorporating the missingness pattern as a main effect along with the interaction term for Group and the time point to assess the impact of missingness of data on the outcome variables. The results indicate no significant association between the missingness of data and the outcome variables, supporting the assumption that the missing data was most likely MAR. Moreover, the results of fixed effects also remained similar to that of the primary approach.

**Table S7.** Sensitivity analysis results (Time*Group results).

| **Model 1:** ***Imputation Model method = "pmm"*** | | | | | | | |
|---|---|---|---|---|---|---|---|
| **Term** | ***Estimate, B (CI)** | ***Pooled SE** | **t-Statistic** | **df** | **p-Value** | **Bonferroni-adjusted p-value** | **Cohen *d* (SE)** |
| **GAD-7** | | | | | | | |
| Week 0 (Baseline) | 0.10 (-1.43, 1.64) | 0.778 | 0.134 | 279.993 | 0.894 | 1.000 | 0.03 (0.046) |
| Week 5 | -2.27 (-4.23, -0.31) | 0.99 | -2.298 | 117.24 | 0.023 | 0.187 | -0.58 (0.047) |
| Week 10 | -3.71 (-5.67, -1.75) | 0.991 | -3.748 | 136.859 | 0.00026 | 0.002** | -0.92 (0.048) |
| Follow-up | -3.88 (-5.91, -1.85) | 1.024 | -3.792 | 113.771 | 0.00024 | 0.002** | -0.95 (0.048) |
| **BAI** | | | | | | | |

| | | | | | | | |
|---|---|---|---|---|---|---|---|
| Week 0 (Baseline) | -0.58 (-4.62, 3.45) | 2.05 | -0.285 | 279.993 | 0.776 | 1 | -0.06 (0.046) |
| Week 5 | -4.24 (-9.10, 0.61) | 2.44 | -1.737 | 85.948 | 0.086 | 0.687 | -0.46 (0.046) |
| Week 10 | -7.50 (-12.19, -2.81) | 2.37 | -3.163 | 133.543 | 0.00193 | 0.015* | -0.82 (0.048) |
| Follow-up | -7.67 (-12.47, -2.87) | 2.43 | -3.161 | 120.611 | 0.00199 | 0.016* | -0.87 (0.048) |
| **PSWQ** | | | | | | | |
| Week 0 (Baseline) | 1.58 (-0.54, 3.71) | 1.08 | 1.464 | 279.993 | 0.144 | 1 | 0.34 (0.046) |
| Week 5 | -3.95 (-6.44, -1.45) | 1.27 | -3.118 | 212.921 | 0.002 | 0.017 | -0.47 (0.046) |
| Week 10 | -4.60 (-7.56, -1.65) | 1.49 | -3.1 | 86.617 | 0.0026 | 0.021 | -0.61 (0.047) |
| Follow-up | -3.02 (-5.82, -0.22) | 1.41 | -2.136 | 114.161 | 0.035 | 0.278 | -0.23 (0.046) |
| **HADS-A** | | | | | | | |
| Week 0 (Baseline) | 0.25 (-1.26, 1.76) | 0.77 | 0.326 | 279.993 | 0.745 | 1 | 0.08 (0.046) |
| Week 5 | -1.51 (-3.21, 0.19) | 0.86 | -1.755 | 159.246 | 0.081 | 0.65 | -0.34 (0.046) |
| Week 10 | -2.84 (-4.67, -1.01) | 0.93 | -3.07 | 170.122 | 0.0025 | 0.02 | -0.70 (0.047) |
| Follow-up | -2.19 (-4.04, -0.34) | 0.94 | -2.335 | 173.441 | 0.021 | 0.166 | -0.45 (0.046) |
| **HADS-D** | | | | | | | |
| Week 0 (Baseline) | 1.19 (-0.48, 2.85) | 0.85 | 1.405 | 279.993 | 0.161 | 1 | 0.32 (0.046) |
| Week 5 | -1.42 (-3.20, 0.36) | 0.9 | -1.581 | 158.163 | 0.116 | 0.928 | -0.06 (0.046) |
| Week 10 | -2.66 (-4.77, -0.56) | 1.06 | -2.513 | 82.141 | 0.0139 | 0.111 | -0.35 (0.046) |
| Follow-up | -2.46 (-4.42, -0.49) | 0.99 | -2.47 | 119.984 | 0.0149 | 0.119 | -0.30 (0.046) |

**Unstandardized Estimates and SE*

| **Model 2:** ***LMM model (Fixed Effect - Group, time; Random intercepts: subject-level (PatientID), time-level*** | | | | | | | |
|---|---|---|---|---|---|---|---|
| **Term** | ***Estimate, B (CI)** | ***Pooled SE** | **t-Statistic** | **df** | **p-Value** | **Bonferroni-adjusted p-value** | **Cohen *d* (SE)** |
| **GAD-7** | | | | | | | |
| Week 0 (Baseline) | 0.104 (−1.306 to 1.514) | 0.716 | 0.145 | 279.993 | 0.884 | 1.0 | 0.030 (0.046) |
| Week 5 | -2.274 (−4.328 to -0.220) | 1.038 | -2.19 | 129.328 | 0.03* | 0.242 | -0.578 (0.047) |

| | | | | | | | |
|---|---|---|---|---|---|---|---|
| Week 10 | -3.714 (−5.650 to -1.777) | 0.979 | -3.793 | 133.852 | <0.001*** | 0.0018 ** | -0.917 (0.048) |
| Follow-up | -3.882 (−5.836 to -1.929) | 0.985 | -3.941 | 104.299 | <0.001*** | 0.0012 ** | -0.953 (0.048) |
| **BAI** | | | | | | | |
| Week 0 (Baseline) | -0.583 (−4.630 to 3.464) | 2.056 | -0.284 | 279.993 | 0.777 | 1.0 | -0.058 (0.046) |
| Week 5 | -4.353 (−8.880 to 0.174) | 2.294 | -1.898 | 174.986 | 0.059 | 0.475 | -0.467 (0.046) |
| Week 10 | -7.882 (−12.111 to -3.654) | 2.143 | -3.678 | 181.172 | 0.0003 | 0.0025 | -0.868 (0.048) |
| Follow-up | -8.081 (−12.585 to -3.578) | 2.284 | -3.538 | 198.271 | 0.0005 | 0.004 | -0.926 (0.048) |
| **PSWQ** | | | | | | | |
| Week 0 (Baseline) | 1.583 (−0.288 to 3.455) | 0.951 | 1.665 | 279.993 | 0.097 | 0.776 | 0.343 (0.046) |
| Week 5 | -4.060 (−6.522 to -1.599) | 1.246 | -3.258 | 158.346 | 0.001 | 0.011 | -0.507 (0.046) |
| Week 10 | -4.764 (−7.239 to -2.288) | 1.245 | -3.826 | 86.304 | 0.0002 | 0.002 | -0.650 (0.047) |
| Follow-up | -3.279 (−6.249 to -0.310) | 1.497 | -2.191 | 99.983 | 0.031 | 0.246 | -0.265 (0.046) |
| **HADS-A** | | | | | | | |
| Week 0 (Baseline) | 0.250 (−1.034 to 1.534) | 0.652 | 0.383 | 279.993 | 0.702 | 1.0 | 0.079 (0.046) |
| Week 5 | -1.579 (−3.292 to 0.134) | 0.866 | -1.824 | 127.658 | 0.07 | 0.564 | -0.359 (0.046) |
| Week 10 | -2.736 (−4.423 to -1.050) | 0.853 | -3.208 | 145.29 | 0.002 | 0.013 | -0.665 (0.047) |
| Follow-up | -2.207 (−4.136 to -0.279) | 0.976 | -2.263 | 142.74 | 0.025 | 0.201 | -0.462 (0.046) |
| **HADS-D** | | | | | | | |
| Week 0 (Baseline) | 1.188 (−0.331 to 2.706) | 0.772 | 1.539 | 279.993 | 0.125 | 0.999 | 0.317 (0.046) |

| | | | | | | | |
|---|---|---|---|---|---|---|---|
| Week 5 | -1.464 (−3.327 to 0.400) | 0.94 | -1.557 | 105.556 | 0.122 | 0.979 | -0.065 (0.046) |
| Week 10 | -2.592 (−4.589 to -0.595) | 1.003 | -2.584 | 77.264 | 0.012 | 0.093 | -0.334 (0.046) |
| Follow-up | -2.478 (−4.583 to -0.373) | 1.061 | -2.336 | 98.996 | 0.021 | 0.172 | -0.299 (0.046) |

*Unstandardized Estimates and SE*

| **Model 3:** *LMM model (Fixed Effect - Group, time; Random intercepts: subject-level (PatientID), time-level* | | | | | | | |
|---|---|---|---|---|---|---|---|
| **Term** | ***Estimate, B (CI)** | ***Pooled SE** | **t-Statistic** | **df** | **p-Value** | **Bonferroni-adjusted p-value** | **Cohen *d* (SE)** |
| **GAD-7** | | | | | | | |
| Week 0 (Baseline) | 0.104 (-1.318 to 1.527) | 0.716 | 0.145 | 94 | 0.885 | 1.0 | 0.030 (0.046) |
| Week 5 | -1.739 (-3.613 to 0.136) | 0.951 | -1.828 | 225 | 0.069 | 0.551 | -0.457 (0.207) |
| Week 10 | -3.753 (-5.547 to -1.958) | 0.911 | -4.121 | 225 | 5.3E-05 | 0.00042 | -0.992 (0.216) |
| Follow-up | -3.248 (-5.004 to -1.492) | 0.891 | -3.645 | 225 | 0.00033 | 0.0027 | -0.855 (0.213) |
| **BAI** | | | | | | | |
| Week 0 (Baseline) | -0.583 (-4.665 to 3.499) | 2.056 | -0.284 | 94 | 0.777 | 1.0 | -0.058 (0.204) |
| Week 5 | -3.866 (-7.920 to 0.189) | 2.058 | -1.879 | 226 | 0.062 | 0.493 | -0.416 (0.206) |
| Week 10 | -7.898 (-12.015 to -3.781) | 2.089 | -3.78 | 226 | 0.0002 | 0.0016 | -0.793 (0.212) |
| Follow-up | -8.138 (-12.527 to -3.749) | 2.227 | -3.654 | 226 | 0.00032 | 0.0026 | -0.892 (0.214) |
| **PSWQ** | | | | | | | |
| Week 0 (Baseline) | 1.583 (-0.304 to 3.471) | 0.951 | 1.665 | 94 | 0.099 | 0.793 | 0.340 (0.206) |
| Week 5 | -4.089 (-6.376 to -1.801) | 1.161 | -3.522 | 226 | 0.00052 | 0.0041 | -0.492 (0.207) |

| | | | | | | | |
|---|---|---|---|---|---|---|---|
| Week 10 | -4.370 (-6.330 to -2.410) | 0.995 | -4.393 | 226 | 1.7E-05 | 0.00013 | -0.609 (0.209) |
| Follow-up | -2.491 (-5.109 to 0.128) | 1.329 | -1.875 | 226 | 0.062 | 0.497 | -0.170 (0.205) |
| **HADS-A** | | | | | | | |
| Week 0 (Baseline) | 0.250 (-1.045 to 1.545) | 0.652 | 0.383 | 94 | 0.702 | 1.0 | 0.079 (0.205) |
| Week 5 | -1.767 (-3.191 to -0.342) | 0.723 | -2.443 | 226 | 0.015 | 0.12 | -0.404 (0.206) |
| Week 10 | -2.926 (-4.446 to -1.406) | 0.771 | -3.793 | 226 | 0.00019 | 0.0015 | -0.671 (0.210) |
| Follow-up | -2.145 (-3.980 to -0.310) | 0.931 | -2.303 | 226 | 0.022 | 0.178 | -0.357 (0.206) |
| **HADS-D** | | | | | | | |
| Week 0 (Baseline) | 1.188 (-0.345 to 2.720) | 0.772 | 1.539 | 94 | 0.125 | 1.0 | 0.317 (0.205) |
| Week 5 | -1.059 (-2.564 to 0.445) | 0.763 | -1.388 | 226 | 0.166 | 1.0 | -0.278 (0.205) |
| Week 10 | -2.185 (-3.811 to -0.559) | 0.825 | -2.648 | 226 | 0.0086 | 0.069 | -0.279 (0.205) |
| Follow-up | -2.092 (-3.881 to -0.303) | 0.908 | -2.304 | 226 | 0.022 | 0.177 | -0.255 (0.205) |

**Unstandardized Estimates and SE*

| ***Model 4: Pattern-Mixture Model (Pattern Baseline: Completers)*** | | | | | |
|---|---|---|---|---|---|
| **Term** | ***Estimate, B (CI)** | ***Pooled SE** | **t-Statistic** | **p-Value** | **Bonferroni-adjusted p-value** |
| **GAD-7** | | | | | |
| Week 0 (Baseline) | -0.17 [-1.84, 1.5] | 0.85 | -0.21 | 0.837 | 1.0 |
| Week 5 | -1.60 [-3.37, 0.18] | 0.9 | -1.77 | 0.077 | 0.77 |
| Week 10 | -3.67 [-5.45, -1.89] | 0.91 | -4.06 | <0.001 | 0.0006 |
| Follow-up | -3.19 [-4.97, -1.41] | 0.91 | -3.52 | 0.0005 | 0.005 |
| Pattern: Dropout | 0.76 [-1.28, 2.8] | 1.03 | 0.73 | 0.464 | 1.0 |

| | | | | | |
|---|---|---|---|---|---|
| Pattern: Intermittent Missing | 0.99 [-3.39, 5.37] | 2.21 | 0.45 | 0.655 | 1.0 |
| **BAI** | | | | | |
| Week 0 (Baseline) | -1.19 [-5.59, 3.21] | 2.23 | -0.53 | 0.595 | 1.0 |
| Week 5 | -3.82 [-7.86, 0.21] | 2.05 | -1.86 | 0.063 | 0.63 |
| Week 10 | -7.93 [-12.01, -3.84] | 2.07 | -3.82 | 0.00017 | 0.0017 |
| Follow-up | -8.19 [-12.28, -4.11] | 2.07 | -3.95 | 0.0001 | 0.001 |
| Pattern: Dropout | 0.41 [-4.96, 5.78] | 2.71 | 0.15 | 0.88 | 1.0 |
| Pattern: Intermittent Missing | 11.4 [-0.87, 23.68] | 6.18 | 1.84 | 0.068 | 0.68 |
| **PSWQ** | | | | | |
| Week 0 (Baseline) | 1.42 [-0.86, 3.7] | 1.16 | 1.23 | 0.221 | 1.0 |
| Week 5 | -4.04 [-6.43, -1.65] | 1.21 | -3.32 | 0.001 | 0.01 |
| Week 10 | -4.27 [-6.7, -1.85] | 1.23 | -3.47 | 0.0006 | 0.006 |
| Follow-up | -2.45 [-4.87, -0.03] | 1.23 | -1.99 | 0.048 | 0.477 |
| Pattern: Dropout | 0.41 [-2.34, 3.17] | 1.4 | 0.3 | 0.768 | 1.0 |
| Pattern: Intermittent Missing | 0.83 [-5.16, 6.83] | 3.02 | 0.28 | 0.783 | 1.0 |
| **HADS-A** | | | | | |
| Week 0 (Baseline) | 0.29 [-1.33, 1.92] | 0.82 | 0.36 | 0.721 | 1.0 |
| Week 5 | -1.76 [-3.33, -0.19] | 0.8 | -2.21 | 0.028 | 0.28 |
| Week 10 | -2.96 [-4.55, -1.37] | 0.81 | -3.67 | 0.0003 | 0.003 |

| | | | | | |
|---|---|---|---|---|---|
| Follow-up | -2.2 [-3.79, -0.61] | 0.81 | -2.73 | 0.007 | 0.068 |
| Pattern: Dropout | -0.44 [-2.42, 1.54] | 1.0 | -0.44 | 0.661 | 1.0 |
| Pattern: Intermittent Missing | 2.25 [-2.2, 6.69] | 2.24 | 1.0 | 0.318 | 1.0 |
| **HADS-D** | | | | | |
| Week 0 (Baseline) | 0.8 [-1, 2.59] | 0.91 | 0.87 | 0.383 | 1.0 |
| Week 5 | -1 [-2.59, 0.59] | 0.81 | -1.24 | 0.215 | 1.0 |
| Week 10 | -2.12 [-3.73, -0.52] | 0.82 | -2.6 | 0.01 | 0.098 |
| Follow-up | -2.03 [-3.63, -0.42] | 0.82 | -2.49 | 0.013 | 0.136 |
| Pattern: Dropout | 0.91 [-1.29, 3.11] | 1.11 | 0.82 | 0.415 | 1.0 |
| Pattern: Intermittent Missing | 2.57 [-2.5, 7.65] | 2.56 | 1.01 | 0.317 | 1.0 |

**Unstandardized Estimates and SE*

## 11. Standardized Response Mean (SRM)

**Table S8.** Pre-post treatment outcomes for *Anzeilax* for SRM calculation.

| Pre treatment | Treatment | Control |
|---|---|---|
| n | 48 | 48 |
| Mean | 12.42 | 12.31 |
| SD | 3.57 | 3.45 |

| Post treatment | Treatment | Control |
|---|---|---|
| n | 48 | 48 |
| Mean | 8.83 | 11.04 |
| SD | 3.99 | 4.29 |

| Difference (Post-pre) | Treatment | Control |
|---|---|---|
| n | 48 | 48 |
| Mean | -3.59 | -1.27 |
| SD | 3.80 | 3.94 |

Diff Treatment SD: $\sqrt{\left(3.57^2 + 3.99^2 - (2 * 0.5 * 3.57 * 3.99)\right)} = 3.80$

Diff Control SD: $\sqrt{\left(3.45^2 + 4.29^2 - (2 * 0.5 * 3.45 * 4.29)\right)} = 3.94$

Estimation of Pre-post(difference) SD: $Sp^2 = 3.87$

SRM = $\frac{2.32}{3.87} = 0.5994$

## 12. Impact of Comorbid MDD on GAD Treatment Response

### 1. T-test and effect size (Cohen *d*):

We conducted independent-sample t-tests comparing GAD-7 score change from baseline (week 0) to endpoint (week 10) between treatment participants with and without MDD, separately for the full analysis set (FAS) and per-protocol set (PP). The differences were not statistically significant (FAS: $p = .689$; PP: $p = .486$). However, effect sizes calculating using Cohen *d* suggested medium to large effects in both groups (FAS: $d = 0.74 – 0.83$; PP: $d = 0.79 – 1.15$), indicating meaningful within-group improvements regardless of MDD status.

**Table 1. Group differences in GAD-7 score change by MDD status**

<table>
<tr><td>Analysis set</td><td>Group</td><td>n</td><td>GAD-7 Change (95% CI)</td><td>P value</td></tr>
<tr><td rowspan="2">FAS</td><td>w/out MDD</td><td>21</td><td rowspan="2">-0.53<br>(-2.12, 3.17)</td><td rowspan="2">.689</td></tr>
<tr><td>w/ MDD</td><td>27</td></tr>
<tr><td rowspan="2">PP</td><td>w/out MDD</td><td>10</td><td rowspan="2">-1.39<br>(-2.67, 5.44)</td><td rowspan="2">.486</td></tr>
<tr><td>w/ MDD</td><td>14</td></tr>
</table>

**Table 2. Effect sizes for GAD symptom changes in participants with and without comorbid MDD**

<table>
<tr><td rowspan="2">Analysis set</td><td rowspan="2">Group</td><td colspan="2">Mean</td><td rowspan="2">Mean Change (95% CI)</td><td rowspan="2">Cohen d</td></tr>
<tr><td>Pre</td><td>Post</td></tr>
<tr><td rowspan="2">FAS</td><td>w/out MDD</td><td>11.86</td><td>8.57</td><td>-3.29<br>(-5.31, -1.26)</td><td>0.74<br>(0.25, 1.22)</td></tr>
<tr><td>w/ MDD</td><td>12.85</td><td>9.04</td><td>-3.81<br>(-5.63, -2.00)</td><td>0.83<br>(0.39, 1.27)</td></tr>
<tr><td rowspan="2">PP</td><td>w/out MDD</td><td>11.50</td><td>7.60</td><td>-3.90<br>(-7.41, -0.39)</td><td>0.79<br>(0.06, 1.50)</td></tr>
<tr><td>w/ MDD</td><td>12.43</td><td>7.14</td><td>-5.29<br>(-7.93, -2.64)</td><td>1.15<br>(0.46, 1.82)</td></tr>
</table>

### 2. Linear regression (GAD_Change ~ GAD-7(Week 0) + MDD):

We also fitted linear regressing models within the treatment group, using baseline GAD-7 score (GAD1) and MDD status as predictors of GAD-7 score change. In both FAS and PP analyses, MDD status was not a significant predictor of GAD-7 improvement (FAS: $p = .907$; PP: $p = .726$), while baseline GAD-7 severity (GAD1) was a consistent and significant predictor ($p < .001$ in both sets).

Model:

$$\text{GAD\_change} = \text{MDD.x1} + \text{GAD1.x2}$$

**Table 3. Linear regression analysis predicting GAD-7 change from baseline severity and MDD status**

| Analysis set | Term | Estimate (SE) | *P* value | 95% CI |
| --- | --- | --- | --- | --- |
| FAS | (Intercept) | 4.62 (2.08) | .032 | 0.43, 8.82 |
| | MDD | 0.13 (1.14) | .907 | -2.17, 2.44 |
| | GAD1 | -0.67 (0.16) | <.001 | -0.99, -0.34 |
| PP | (Intercept) | 6.77 (2.74) | .022 | [1.06, 12.47] |
| | MDD | -0.52 (1.48) | .726 | [-3.60, 2.55] |
| | GAD1 | -0.93 (0.22) | <.001 | [-1.38, -0.47] |

**References**


1. Beck AT, Epstein N, Brown G, Steer RA. An inventory for measuring clinical anxiety: psychometric properties. *J Consult Clin Psychol.*. 1988;56(6):893.

2. Bjelland I, Dahl AA, Haug TT, Neckelmann D. The validity of the Hospital Anxiety and Depression Scale: an updated literature review. *J Psychosom Res.*. 2002;52(2):69–77.

3. Dear BF, Titov N, Sunderland M, McMillan D, Anderson T, Lorian C, Robinson E. Psychometric comparison of the generalized anxiety disorder scale-7 and the Penn State Worry Questionnaire for measuring response during treatment of generalised anxiety disorder. *Cogn Behav Ther.*. 2011;40(3):216–227.

4. Gabrio A, Plumpton C, Banerjee S, Leurent B. Linear mixed models to handle missing at random data in trial-based economic evaluations. *Health Econ.*. 2022;31(6):1276–1287.

5. Graham JW, Olchowski AE, Gilreath TD. How many imputations are really needed? Some practical clarifications of multiple imputation theory. *Prev Sci.*. 2007;8(3):206–213.

6. Little RJA. A test of missing completely at random for multivariate data with missing values. *J Am Stat Assoc.*. 1988;83:1198–1202.

7. MATRICS Consensus Cognitive Battery. Imputation procedures for missing data in clinical research. 2014.

8. Molded-Case Circuit Breaker (MCCB) Marking Guide. UL LLC; Northbrook, IL, USA; 2014.

9. Rubin DB. *Multiple Imputation for Nonresponse in Surveys*. New York, NY: John Wiley & Sons Inc.; 1987.

10. Spitzer RL, Kroenke K, Williams JB, Löwe B. A brief measure for assessing generalized anxiety disorder: the GAD-7. *Arch Intern Med.*. 2006;166(10):1092–1097.

11. van Buuren S, Groothuis-Oudshoorn CGM. mice: Multivariate Imputation by Chained Equations in R. *J Stat Softw.*. 2011;45(3). Available at: http://www.jstatsoft.org/v45/i03.

12. West BT, Galecki AT. An Overview of Current Software Procedures for Fitting Linear Mixed Models. *Am Stat.*. 2012;65(4):274–282.
